# Unjustified Poisson assumptions lead to overconfident estimates of the effective reproductive number

**DOI:** 10.1017/S0950268826101605

**Published:** 2026-05-25

**Authors:** Barbora Němcová, Isaac H. Goldstein, Jessalyn Sebastian, Volodymyr M. Minin, Johannes Bracher

**Affiliations:** 1 Institute of Statistics, Karlsruhe Institute of Technology, Germany; 2 Helmholtz Information & Data Science School for Health, Helmholtz Association Helmholtz Information & Data Science Academy, Germany; 3 Department of Statistics, Stanford University, USA; 4 Department of Statistics, University of California Irvine, USA; 5 Heidelberg Institute for Theoretical Studies, Germany

**Keywords:** Infectious disease epidemiology, Effective reproductive number, Uncertainty quantification, Renewal equation, Poisson distribution

## Abstract

Time-varying effective reproductive numbers of infectious diseases are commonly estimated using renewal equation models. In the widely applied R package EpiEstim and various related tools, this approach is combined with a Poisson distributional assumption. This has been criticized on various occasions, mostly on grounds of general model realism or a desire to estimate dispersion parameters. Here, we argue that an important issue arising from the Poisson assumption is that inference about the effective reproductive number becomes overconfident in the presence of overdispersion. The extent to which standard errors are underestimated follows from theory on generalized linear models in a straightforward manner. We therefore recommend to replace the Poisson assumption by quasi-Poisson or negative binomial extensions, and contrast their respective properties. We illustrate our arguments in detailed simulation studies and three case studies on Ebola, pandemic influenza, and COVID-19.

## Introduction

The effective reproductive number, defined as the expected number of new infections generated by a single infectious individual in a population that is no longer fully susceptible [1], is a key quantity characterizing disease outbreak dynamics. It is commonly estimated using renewal equation models, often combined with Poisson distributional assumptions. This approach was popularized by the R package EpiEstim [2], which due to its broad applicability, ease of use, and thorough documentation has become a standard tool [3]. It has also been integrated in numerous methodological extensions (e.g., [4–6]) and alternative R packages including EpiInvert [7], ern [8], estimateR [9], and most recently Rt.GLM [10]. In this article, we are concerned with the uncertainty surrounding estimated reproductive numbers based on the renewal equation and a Poisson assumption for the observed incidence. We demonstrate that the associated standard errors are underestimated in the presence of overdispersion, a common feature of infectious disease surveillance data. Especially when reported incidence is high, uncertainty intervals based on Poisson assumptions get vanishingly narrow, overstating the certainty of the inference. While this has been pointed out empirically [11–13], we provide a concise summary of relevant statistical properties. We moreover contrast several methods to account for overdispersion, which turn out to impact not only estimated standard errors, but also point estimates.

Transmission heterogeneity, superspreading and overdispersion are widely studied phenomena in the epidemic modelling literature. A number of recent contributions have been made with respect to renewal equation modelling [14–17]. Many of them, most explicitly Zhang et al. and Pakkanen et al. [18, 19], link this to branching process models and the seminal work by Lloyd-Smith [20]. Branching processes accommodate individual-level heterogeneity in a very natural way, and with specific distributional choices can be shown to be equivalent to renewal equation models [18].

Gostic et al. [1, p. 3] argue that appropriate confidence intervals rather than mere point estimates are needed to assess whether the effective reproductive number has crossed the threshold value of one, and discuss various sources of uncertainty. The point of our paper is that even the best understood source of uncertainty – classic statistical estimation uncertainty – is handled insufficiently by many commonly used methods, and discuss the necessary remedies. This matters when estimating and interpreting reproductive numbers is the primary goal, but also when estimates feed into multi-step analyses. Overconfidence may then permeate into the downstream parts (see [21] for a general discussion). In renewal equation-based forecasting, it is therefore common to propagate uncertainty about the reproductive number using a multiple imputation approach [22]. This seems to be less common in regression modelling exercises linking estimated reproductive numbers to intervention measures, with, for example, Li and colleagues [23] ignoring the associated estimation uncertainty. However, as pointed out, for example, by Lison et al. [24], this neglects part of the uncertainty and may lead to unwarranted conclusions about the effectiveness of intervention measures. The underlying statistical issues have been discussed in detail in the econometric literature on *generated outcome variables* [25].

The remainder of the paper outlines the challenges arising from the presence of overdispersion in the classic Poisson renewal equation model for estimating the effective reproductive number, along with possible model extensions. We illustrate our arguments using simulations and case studies on COVID-19, Ebola, and pandemic influenza.

## Methods

### The Poisson renewal equation model

While the effective reproductive number can be defined in multiple ways [26], we focus on the *instantaneous* effective reproductive number, which is considered particularly suitable for real-time monitoring [1]. It is defined as the expected number of secondary infections occurring at time t that are generated by individuals infected before t , with each infective’s contribution weighted by their relative infectiousness at time t . The effective reproductive number is often denoted by $R_{e}$ or $R_{t}$ , as its variation over time is what makes real-time estimation relevant. However, in EpiEstim and related approaches, this temporal variation is not modelled explicitly. Instead, the reproductive number is assumed to be constant within estimation windows, usually of length T=7 days. We hence drop the index t and write simply R , while indexing the days in the estimation window by t=1,…,T . It is implicitly understood that the estimation exercise will be repeated for a set of rolling time windows, leading to a trajectory of estimates.

Daily or weekly incidence counts $X_{t},t=1,\ldots ,T$ are linked to R via the renewal equation [27] and a Poisson assumption,

(1) $$X_{t}|X_{t-1},\ldots ,X_{0}∼\mathrm{Pois}\left(\mu_{t}\right),\;\mu_{t}=R\times \Lambda_{t},\;\Lambda_{t}=\sum_{d=1}^{D}w_{d}\times X_{t-d}.$$

Here, $\mu_{t}$ is the expectation of the Poisson distribution, while the probabilities $w_{1},\ldots ,w_{D}$ represent a known discrete-time serial interval distribution. Serial intervals are expressed in the same time steps as incidence counts, and are assumed to have a maximum possible value of D . We note that the distributional assumption holds conditionally on past values $X_{t-1}$ , … , $X_{0}$ , on which $X_{t}$ depends through $\Lambda_{t}$ (see Web Appendix 1 of [2]). The Poisson distribution is chosen as it makes Bayesian inference straightforward without the need for Monte Carlo methods [2].

Throughout the paper, we refer to $w_{1},\ldots ,w_{D}$ as the distribution of the serial interval (the time between the onset of symptoms in a pair of consecutive cases) rather than the generation time (the time between two consecutive infections), as the exact times of infection are typically unobserved. It is known that using the serial interval distribution, which has a larger variance than the generation time distribution, introduces some light bias in the estimation of reproductive numbers [28]. However, this has become standard practice [2], and we do not explore this aspect in detail.

Equation (1) can be read as a Poisson regression with an identity link, no intercept and $\Lambda_{t}$ as the sole covariate [12, 13]. The properties of the resulting estimates can hence be studied in the framework of generalized linear regression [29]. This is most straightforward for maximum likelihood (ML) estimates; the results, however, also translate to the Bayesian approach by [2], which behaves similarly unless incidence is very low (Supplement B.1.2). The ML estimator takes the intuitive form

(2) $${\hat{R}}_{\mathrm{Po}}=\frac{\sum_{t=1}^{T}X_{t}}{\sum_{t=1}^{T}\Lambda_{t}},$$

where the index ‘Po’ indicates the underlying distributional assumption. The estimated standard error

(3) $$\hat{\mathrm{se}}\left({\hat{R}}_{\mathrm{Po}}\right)=\frac{{\hat{R}}_{\mathrm{Po}}}{\sqrt{\sum_{t=1}^{T}X_{t}}}$$

depends on ${\hat{R}}_{\mathrm{Po}}$ and the total incidence over the estimation window, but not the variability of the time series conditioned on past values. This is a consequence of the equidispersion property of the Poisson distribution, which implies $\sigma_{t}^{2}=\mu_{t}$ , with $\sigma_{t}^{2}$ the conditional variance $Var(X_{t}\;|X_{t-1},\ldots ,X_{0})$ . Derivations of equations (2) and (3) are provided in Supplement B.1.1, where we also discuss the challenges due to the dynamic nature of model (1).

The estimated standard error (3) can be used to derive Wald-type confidence intervals, which for a confidence level of 95% are given by ${\hat{R}}_{\mathrm{Po}}\pm 1.96\times \hat{\mathrm{se}}\left({\hat{R}}_{\mathrm{Po}}\right)$ . Empirical coverage fractions of such intervals in simulation studies are a straightforward way to assess uncertainty quantification under varying conditions. We will address this in Section ‘Simulation study’.

### Overdispersion and how to account for it

#### Impact on the Poisson estimator

In practice, infectious disease incidence data usually display overdispersion relative to the Poisson model. Throughout the paper, we conceive this as conditional variances exceeding conditional means, $\sigma_{t}^{2}>\mu_{t}$ . Overdispersion may be due to a variety of factors, including superspreading events [20] and incomplete reporting [30]. The Poisson assumption will then cause our inference for R to be overconfident; see Supplementary Section A for an intuitive illustration. Well-known theory on generalized linear regression [29, Sec. 5.5] implies that expression (3) underestimates the true standard error of ${\hat{R}}_{\mathrm{Po}}$ by a factor of $\sqrt{\phi }$ , with

(4) $$\phi =\frac{\sum_{t=1}^{T}\sigma_{t}^{2}}{\sum_{t=1}^{T}\mu_{t}}.$$

The *variance inflation factor* ϕ depends on the relationship between $\mu_{t}$ and $\sigma_{t}^{2}$ . We note that equation (4) holds irrespective of the exact form of this relationship, even though in practice one usually makes more specific assumptions (see the next section). As we shall see in Section ‘Illustration for three outbreaks’, ϕ may be quite large in practice, meaning that standard errors estimated under the Poisson assumption may be considerably too small.

Translating equation (4) to the width of the resulting confidence interval, a (1−α) confidence interval from the Poisson renewal equation model has coverage probability

(5) $$1-2\times \Phi \left[\Phi^{-1}\left(\frac{\alpha }{2}\right)/\sqrt{\phi }\right],$$

where Φ is the cumulative distribution function of the standard normal distribution. This matches the intended level (1−α) only if ϕ=1 , and decreases if ϕ>1 . The derivation of the coverage probability (5) is provided in Supplement B.2.2.

In the following, we will outline three options to account for overdispersion, all of which have been applied in the literature. While this is typically motivated by the desire to estimate dispersion parameters [16, 18] and general model realism [31], our focus is on uncertainty quantification.

#### Quasi-Poisson regression

Quasi-Poisson regression, used by [13], serves to correct the uncertainty quantification for the Poisson model by estimating the variance inflation factor ϕ from equation (4). Assuming a linear relationship between $\mu_{t}$ and $\sigma_{t}^{2}$ , it can be estimated from the residuals of the Poisson model (see Supplement B.2.1). The corrected standard error is then obtained by multiplying expression (3) with $\sqrt{\hat{\phi }}$ . We note that quasi-Poisson regression is not a full probability model, making it unsuitable, for example, for likelihood-based model comparisons and for sampling future trajectories.

#### Negative binomial regression

The most common full probability model to generalize the Poisson assumption is the negative binomial distribution. We here parameterize it by its mean and a dispersion parameter, which, for example, in the R software is called the ‘size parameter’. Despite its name, a higher value of the dispersion parameter indicates lower dispersion (see below for formulas), and the Poisson distribution is the limiting case of an infinite dispersion parameter. The dispersion parameter can be handled in two ways ([32], p.73), which we call *NegBin-L* and *NegBin-Q*, as they imply *linear* and *quadratic* mean–variance relationships. Irrespective of which is used, estimates and standard errors for R are obtained using standard regression methodology and naturally account for overdispersion.

##### NegBin-L

In the NegBin-L renewal equation (see, e.g., [16, 18]),

(6) $$X_{t}|X_{t-1},\ldots ,X_{0}∼NegBin(mean=\mu_{t},disp=\xi \times \mu_{t}),$$

the dispersion parameter is assumed to be proportional to the mean. This implies a linear mean–variance relationship $\sigma_{t}^{2}=\left(1+1/\xi \right)\times \mu_{t}$ as in the quasi-Poisson model, and can be motivated by overdispersed individual offspring distributions [18]. While some differences between NegBin-L and quasi-Poisson regression exist [33], in our applications we found similar behaviour, with point estimates almost identical to those from equation (2).

##### NegBin-Q

We therefore focus primarily on NegBin-Q, defined as

(7) $$X_{t}|X_{t-1},\ldots ,X_{0}∼NegBin(mean=\mu_{t},disp=\psi ),$$

and implying a quadratic mean–variance relationship $\sigma_{t}^{2}=\mu_{t}+\mu_{t}^{2}/\psi$ . This version, employed, for example, by [12, 18, 31, 34], can be motivated by temporal variation of R over the estimation window [18]. Despite the mean structure $\mu_{t}=R\times \Lambda_{t}$ mirroring the Poisson version, the ML estimator ${\hat{R}}_{\mathrm{NBQ}}$ is not identical to ${\hat{R}}_{\mathrm{Po}}$ from equation (2). No closed expression exists, but if overdispersion is sufficiently strong, the approximation

(8) $${\hat{R}}_{\mathrm{NBQ}}\approx \frac{1}{T}\times \sum_{t=1}^{T}\frac{X_{t}}{\Lambda_{t}}$$

holds; see derivation in Supplement B.3.1. Unlike in equation (2), the ratios $X_{1}/\Lambda_{1},\ldots ,X_{T}/\Lambda_{T}$ thus contribute equally to the estimate. This is because they are all equally informative under a quadratic mean–variance relationship, irrespective of the magnitude of $\Lambda_{t}$ and $X_{t}$ (see discussion in [35]). This also explains why the approximate standard error

(9) $$\hat{\mathrm{se}}\left({\hat{R}}_{\mathrm{NBQ}}\right)\approx {\hat{R}}_{\mathrm{NBQ}}\times \sqrt{{\left(\hat{\psi }\times T\right)}^{-1}}$$

features the number of observations T rather than the total incidence as in equation (3). It moreover depends on the estimated dispersion parameter $\hat{\psi }$ , which reflects the conditional variability of the time series and can be computed from the model residuals (Supplement B.3). It generally holds that $\hat{\mathrm{se}}({\hat{R}}_{\mathrm{NBQ}})\geq \hat{\mathrm{se}}({\hat{R}}_{\mathrm{Po}})$ , that is, the estimated standard error is at least equal to its Poisson counterpart. The approximations (8) and (9) are assessed empirically in Supplementary Figure S22.

ML estimation in the negative binomial renewal equation models (6) and (7) can run into convergence issues if the data used for fitting show no or very little overdispersion. This is because the dispersion parameter ξ in the NegBin-L model (or ψ in the NegBin-Q) is usually estimated on a log-transformed scale to ensure positivity. If the highest likelihood is achieved for a very high value of ξ , the likelihood as a function of log(ξ) becomes almost flat for large log(ξ) . This causes numerical failure in the computation of point estimates and/or standard errors. Whenever this occurs, we revert to the Poisson model (1), and report the corresponding point estimates and standard errors. In such low-dispersion settings, reverting to the Poisson is numerically convenient and statistically appropriate, as the Poisson distribution can be understood as the limiting form of the negative binomial when overdispersion vanishes.

Quasi-Poisson regression has certain advantages over the negative binomial versions if the mean–variance relationship is mis-specified [36]. In our setting, however, both equations (2) and (8) remain unbiased and consistent in this case, and only their efficiency is reduced. How strongly the two estimators differ is an empirical question we will return to in Section ‘Illustration for three outbreaks’.

##### Comparing model fits and testing for the presence of conditional overdispersion

To assess whether and to what degree the negative binomial models improve the fit relative to the Poisson, we compare the AIC values and employ likelihood ratio (LR) tests. The quasi-Poisson model cannot be included in this comparison, as it does not have a proper likelihood function. In the LR test, we test the null hypothesis of no conditional overdispersion. Specifically, we test $H_{0}:1/\xi =0$ (NegBin-L) and $H_{0}:1/\psi =0$ (NegBin-Q) against one-sided alternatives. To account for the fact that the null hypotheses are on the border of the parameter space we employ the correction suggested by Molenberghs and Verbeke [37].

### Setup of the simulation study

To support our claim that Poisson assumptions lead to overconfidence in the estimation of R we conduct a simulation study. We combine different distributional assumptions for the data-generating process and the model used for R estimation, and compute empirical coverage fractions of Wald confidence intervals. These are given by the fraction of cases in which confidence intervals with a given nominal level contain the true reproductive number R .

In the main manuscript, we focus on time series generated from the NegBin-L renewal equation (6). We generate 1,000 trajectories from each of four scenarios, which are defined by a combination of two parameters: two values of the reproductive number R∈{1.5,2.5} and two levels of overdispersion ξ∈{2,0.2} . For all scenarios, we used the same shifted gamma serial interval distribution with a mean of 7.5 days and a standard deviation of 2.1 days, cut off at D=14 days. All trajectories are initialized by the same sequence of values (2 , 7 , 6 , 4 , 3 , 7 , 7 , 6 , 6 , 6 , 4 , 9 , 3 , 7) . In Supplementary Section C.2.1, we include additional simulation scenarios, where the trajectories are initialized by higher values around 100 in order to produce different trajectories with overall higher incidence. After this deterministic initialization, we generate random trajectories of length 28 days. The first 14 days serve to avoid dependence on the specific initialization sequence (‘burn-in period’), while the last 14 days are used to estimate the value of R . We emphasize that the true R is a constant throughout the 28 days, such that in terms of the mean structure the assumptions shared by all considered models are fulfilled and the theoretical arguments from the previous sections apply. While in reality, smooth variation of R would be expected, our simulation study is concerned with the behaviour of EpiEstim-like methods if all assumptions other than the Poisson distribution are fulfilled. It therefore inherits the constraint of time-constant R values. An adapted simulation where the reproductive number varies smoothly over time is available in Supplementary Section C.2.3.

The serial interval is motivated by the estimate [38] provide for respiratory syncytial virus (RSV; see also [39]). In Supplementary Section C.2.2, we provide further simulation scenarios with serial intervals that are motivated by influenza (mean 3.7 days, sd 2.1 days [40]) and measles (mean 13.7 days, sd 1.5 days [41]).

For each simulated trajectory, R values were estimated under the four distributional assumptions discussed in Section ‘Overdispersion and how to account for it’. The Poisson and quasi-Poisson models were fitted using the stats::glm function in R [42], while gamlss::gamlss [43] was employed for the negative binomial models. To assess the impact of small sample biases, we applied two different estimation window lengths (7 and 14 days) across all scenarios. As described in Section ‘Negative binomial regression’, in cases of convergence issues due to low degrees of observed overdispersion, we reverted from the negative binomial models to the Poisson version.

To make our assessment comprehensive, we ran corresponding simulation studies using the NegBin-Q and Poisson versions of the model as the data-generating processes. In addition, and similarly to [44], we study a setting where none of the considered models matches the data-generating process. To this end, we simulated time series from an overdispersed branching process model. Details on all of these settings are provided in Supplementary Section C.

## Results

### Simulation study

**Figure 1.**
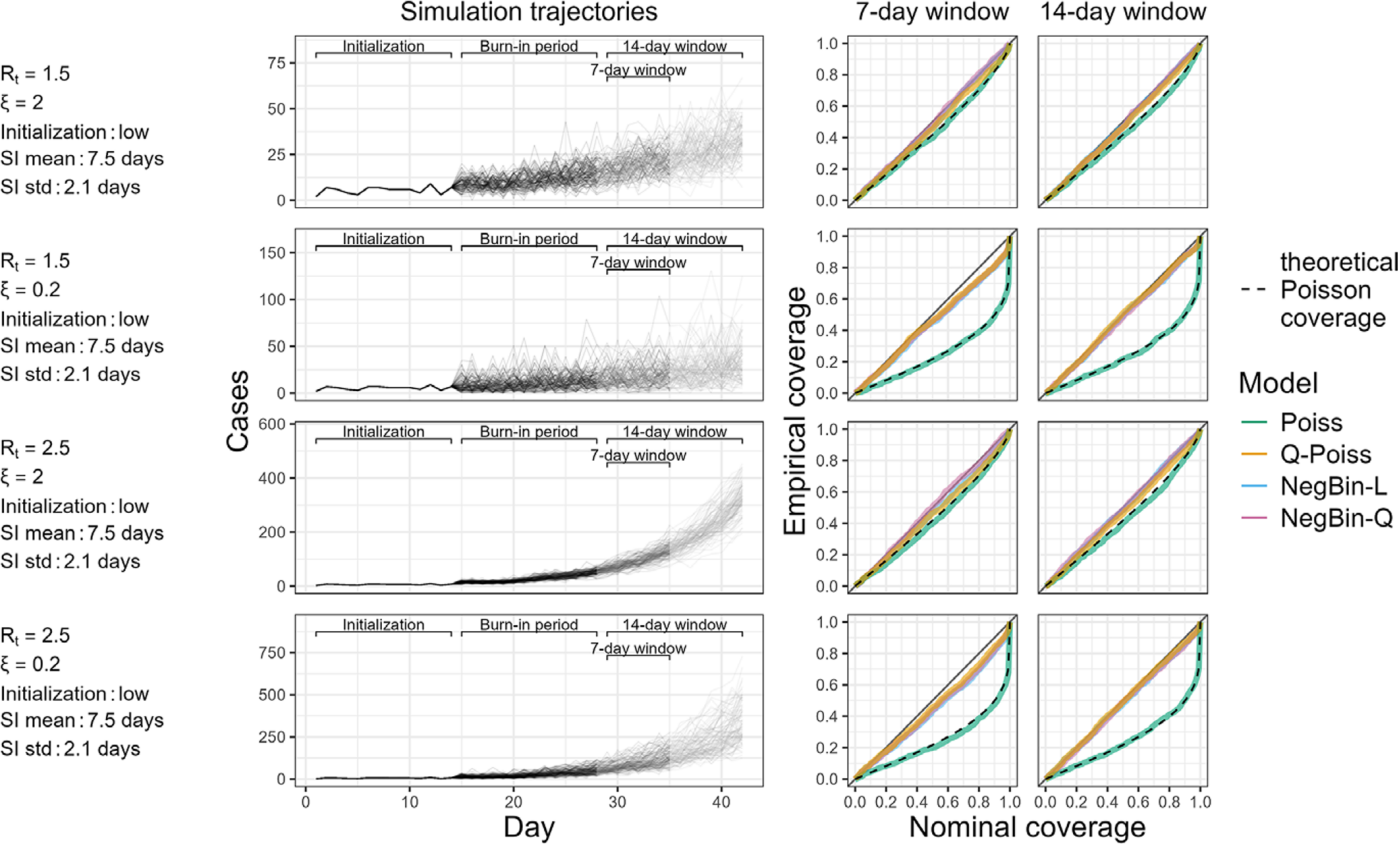
Simulation trajectories generated by the NegBin-L model with a serial interval typical to RSV and empirical coverage of the four models in scenarios initialized by low values (see Supplementary Figure S3 for corresponding results with higher initial values). The left panel shows 1,000 incidence trajectories generated from the renewal equation with the NegBin-L distribution across four scenarios. These are defined by different parameter combinations specified on the left margin of the figure. The right panels display empirical coverage of the true R for Poisson, quasi-Poisson and negative binomial models across nominal coverage levels, using 7-day (middle column) and 14-day (right column) estimation windows. Dashed black lines indicate our theoretical expectation for the coverage of the Poisson model based on equation (5). Figure 1. long description.

Figure 1 shows the sampled trajectories in the different NegBin-L scenarios (left column), along with how often confidence intervals produced under different distributional assumptions cover the true value of R (right column). These coverage proportions are shown for the two window lengths (7 and 14 days) and as a function of the nominal coverage level (1−α) . Perfect calibration corresponds to (1−α)×100 % confidence intervals covering the true value of R in (1−α)×100 % of cases for all 1≤α≤1 . The plotted curve should therefore closely follow the diagonal. Values above indicate overcoverage, while values under the diagonal point to undercoverage and hence overconfidence.

**Figure 2.**
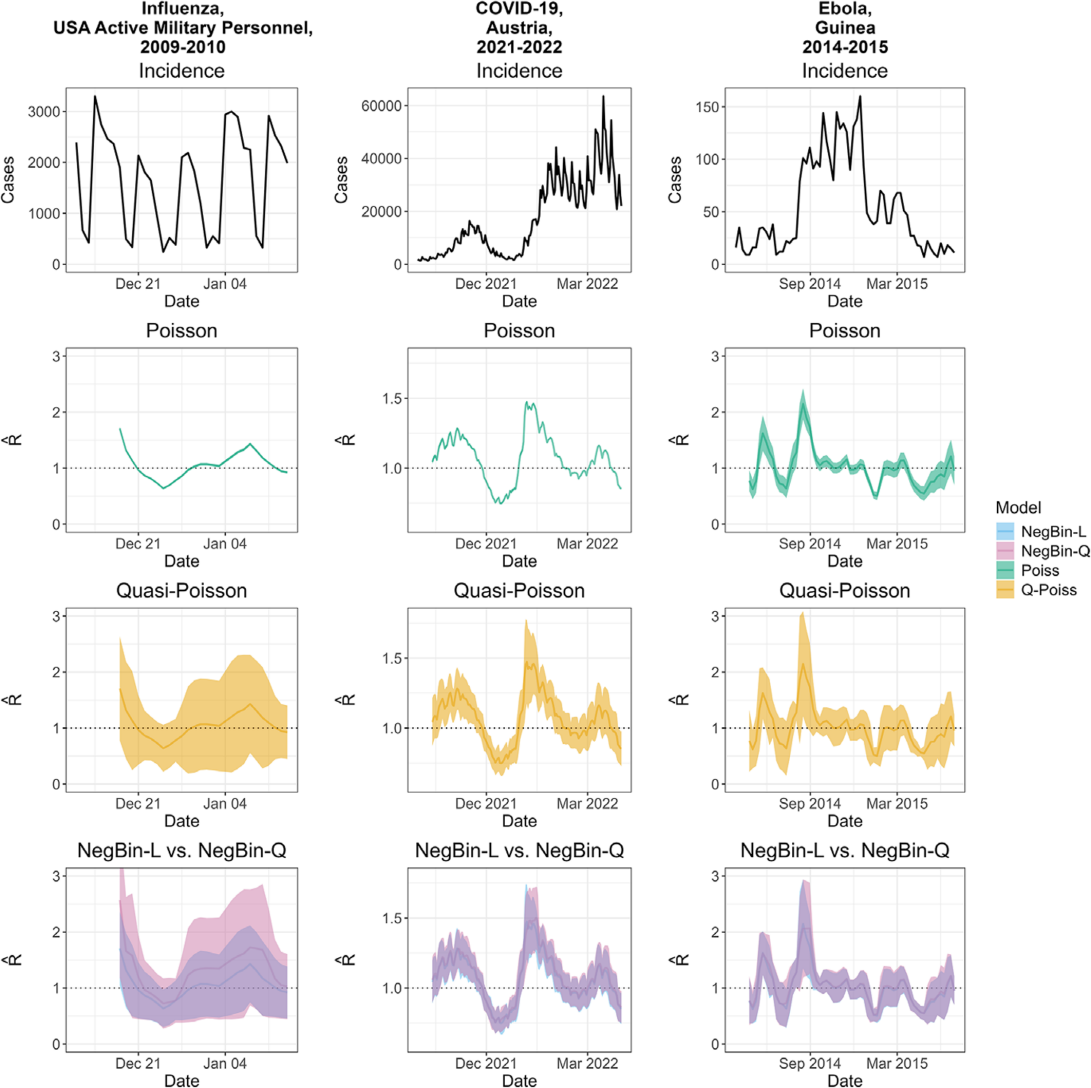
Incidence and estimated effective reproductive numbers for three outbreaks: influenza among active military personnel in the USA, 2009–2010 (left column); COVID-19 in Austria, 2021–2022 (middle); and Ebola in Guinea, 2014–2015 (right). The first row shows case incidences, which are in daily resolution for influenza and COVID-19, and in weekly resolution for Ebola. The second and third rows show the R estimates from the Poisson and quasi-Poisson models, respectively. All estimates are aligned with the last day of the respective estimation window. The fourth row shows a comparison of the R estimates from the NegBin-Q and NegBin-L models. An additional plot overlaying the quasi-Poisson and NegBin-L versions as well as the estimated dispersion parameters are available in Supplementary Figures S20 and S21. Figure 2. long description.

From Figure 1, it is clear that the Poisson model consistently undercovers. The undercoverage is relatively mild in scenarios with low overdispersion (e.g., for R=1.5 , ξ=2 , the 90% confidence interval covers the true value in 82% of the cases), but becomes quite substantial as overdispersion increases. This is especially evident at high nominal coverage levels. For instance, for R=1.5 , ξ=0.2 , the actual coverage of the Poisson model reaches just 50% for a nominal level of 90%. The observed coverage level of the Poisson model is well-approximated by our theoretical expectation based on formula (5), which is shown as a dashed black line. This underscores the validity of our theoretical arguments.

In contrast, the three models accounting for overdispersion generally exhibit similar coverage behaviour with the empirical coverage close to the nominal levels. It thus does not seem crucial whether the mean–variance relationship is specified correctly. A slight tendency to undercover can be spotted for higher nominal coverage levels and larger dispersion values. This issue is less pronounced when the longer 14-day window is used, suggesting the effect of small sample biases when using the 7-day estimation window.

We note that in 18.7% of simulation runs, the fitting algorithm of the negative binomial models did not converge, making it necessary to revert to the Poisson model. In line with our reasoning from Section ‘Negative binomial regression’, this occurred almost exclusively in the scenarios with low overdispersion.

For completeness, we display the empirical distributions of the estimated reproductive number $\hat{R}$ and the associated standard errors in Supplementary Figure S2, as well as the empirical distribution of the dispersion parameter $\hat{\xi }$ , in Supplementary Figure S17. Results when using higher values for initialization, or different serial intervals are similar to the ones discussed above and included in Supplementary Sections C.2.1 and C.2.2, respectively. Simulation scenarios where the reproductive number varies smoothly over time are discussed in Supplementary Section C.2.3, again without any qualitative differences in results. In Supplementary Section C.2.4, we show results for data generated from the NegBin-Q rather than NegBin-L process. Again, it is not crucial whether the mean–variance relationship is specified correctly, and the NegBin-Q, NegBin-L and quasi-Poisson models all perform reasonably. Incidence time series from the Poisson model and associated estimates are shown in Supplementary Section C.2.5. Especially in the high-incidence settings, the resulting bundles of trajectories are very tight, demonstrating that the Poisson distribution in such settings implies an unrealistically low degree of variability. Confidence intervals from both negative-binomial models become somewhat conservative in this setting, a finding we explain in more detail in Section C.2.5 of the Supplement. Finally, Supplementary Section C.2.6 shows results for data generated from a branching process with an overdispersed offspring distribution, instead of the renewal equation model. Again, all qualitative patterns remain unchanged.

### Illustration for three outbreaks

To illustrate the impact of the distributional choice in real-world settings, we present three case studies covering different pathogens and locations. These are based on previous applications of EpiEstim summarized in Table 1. We use shifted gamma serial interval distributions with means and standard deviations retrieved from the respective references. For the influenza and COVID-19 examples, we also use the corresponding estimation window length. For the Ebola data, we present a variation of the analysis from [5]. We aggregate the daily data to weekly totals, rescale the serial interval distribution and employ an estimation window of 4 weeks. This illustrates our point in a setting, where the incidence data are in a weekly resolution and the serial interval is long, so that the times between symptom onsets can likewise be considered in a weekly resolution. As in the simulation study, we show Wald confidence intervals in all cases.

**Table 1.** Summary of the case studies Table 1. long description.

Reference	Disease	Location	Year	Resolution	Serial interval mean (sd)	Estimation window width *T*
[6], data from [45]	Pandemic influenza	US (military personnel)	2009–2010	Daily	3.6 (1.6) days	7 days
[46]	COVID–19	Austria	2021–2022	Daily	3.37 (1.83) days	13 days
[5]	Ebola	Guinea	2014–2015	Weekly	2.19 (1.33) weeks	4 weeks

Figure 2 shows the three incidence time series (top row) and R estimates based on the different distributional assumptions. The second and third rows show the Poisson and quasi-Poisson models, respectively. While the point estimates agree by definition, the Poisson version yields much narrower 95% confidence intervals. For influenza and COVID-19, they are visually indiscernible from the point estimate. For Ebola, the intervals are wider, as suggested by equation (3) for lower incidence counts. Nonetheless, they rarely include R=1.0 , implying high confidence about epidemic growth or decline. The intervals from the quasi-Poisson model (shown in orange), on the other hand, often contain values above and below R=1.0 .

The fourth row overlays results from the NegBin-L model (which behaves similarly to the quasi-Poisson model; Supplementary Figure S21), and the NegBin-Q model. The confidence intervals are comparable for Ebola and COVID-19. For influenza, the NegBin-Q intervals are wider, and there are considerable differences in the point estimates. These are due to the different weighting of the observations in estimators (2) and (8), in conjunction with weekday effects. We discuss this in detail in Supplementary Section A. We note that weekday effects also occur to a lesser degree in the COVID-19 example, but the resulting differences in estimates and confidence intervals are minor.

Ignoring weekday effects can be argued to yield a mis-specified model, and indeed this aspect receives some discussion in [6]. The resulting unexplained variability is absorbed by the dispersion parameters, leading to confidence intervals that may become overly wide. Despite these issues, we kept the influenza example to illustrate the difference between NegBin-L and NegBin-Q, as well as the difficulty of fitting time-homogeneous models to daily data.

In Table 2, we report the average AIC values for the Poisson and negative binomial models across all estimation windows. We additionally calculate the proportion of windows in which the negative binomial versions have lower AIC than the Poisson model, and how often the LR test rejects the null hypothesis of no conditional overdispersion. For COVID-19 and influenza, the average AIC values are drastically lower for the negative binomial models and the window-wise AIC values are lower in every instance. The LR test likewise rejects the null hypothesis in every single case. For Ebola, the differences in AIC are more moderate due to the lower magnitude of the Ebola incidence and the reduced number of observations per window, yet both the AIC comparison and the LR test favour the negative binomial models in more than 75% of the cases. Boxplots of the AIC values are provided in Supplementary Figure S19.

**Table 2.** Summary of the model comparisons using AIC values and the likelihood ratio test to detect conditional overdispersion Table 2. long description.

Disease	Poisson	NegBin-L			NegBin-Q		
	Mean AIC	Mean AIC	% lower AIC than Poisson	% LR-test rejections	Mean AIC	% lower AIC than Poisson	% LR-test rejections
Pandemic Influenza	7,517.84	122.74	100.00	100.00	122.06	100.00	100.00
COVID–19	11,926.48	243.77	100.00	100.00	243.98	100.00	100.00
Ebola	44.73	35.44	79.03	75.81	35.53	77.42	75.81

## Discussion

We argued that unjustified Poisson assumptions lead to overconfident estimation of reproductive numbers and discussed options to account for overdispersion. The quasi-Poisson and NegBin-L versions agreed well in terms of point estimates and uncertainty intervals. The NegBin-Q approach yielded somewhat different results, but in simulation studies, we found that uncertainty quantification was reliable even if the assumed mean–variance relationship was incorrect. In applications to influenza, COVID-19 and Ebola, all three extensions yielded considerably widened uncertainty intervals relative to the Poisson model. The wider intervals included the threshold R=1.0 considerably more often than the Poisson intervals. Differences between estimates from the NegBin-L and quasi-Poisson models on the one hand and the NegBin-Q model on the other hand turned out to be substantial in the case study on influenza. This, however, could be attributed to unaccounted weekday effects, meaning that such discrepancies are indicative of more general model mis-specification issues.

In light of these results, we strongly recommend to account for overdispersion in the estimation of reproductive numbers. While the conjugate prior approach from [2] is difficult to extend, standard regression functions for likelihood-based inference can be adapted. We are aware that an MCMC-based extension of EpiEstim to account for overdispersion in a Bayesian framework is under development, and we recommend it to future users.

Alternative tools like EpiNow2 [47] and EpiLPS [31] already account for overdispersion. EpiNow2 further avoids the need for estimation windows and handles delays between infection events and reporting. This makes estimates more aligned with the actual infection dynamics. Due to its increased complexity, however, EpiNow2 requires more tuning and computational effort, which may present an obstacle to some users. Alternatively, users constrained to renewal equation models with sliding windows can leverage standard regression functionality for quasi-Poisson and negative binomial models. As visible from our accompanying codes (see code availability statement below) and Supplementary Section E, this can be accomplished in a few lines of code.

Our work focuses on correctly quantifying the statistical uncertainty about R . In statistics, there is sometimes a preference to err on the conservative side, and hence prefer too wide over too narrow uncertainty intervals. It could be argued, though, that in a broader decision-making context there is a price to this, as decision-making may be slowed down by overly stringent criteria. A related discussion on converting R estimates and their uncertainty into decision rules can be found in [48].

We conclude by discussing some limitations of our arguments and suggested extensions. By using off-the-shelf generalized linear models, we are deliberately agnostic to the mechanisms behind overdispersion and conflate different sources, such as superspreading, underreporting, and other reporting artefacts. It is thus difficult to assign any mechanistic interpretation to the estimated dispersion parameters, and they cannot serve to derive implications on disease emergence and control [20]. We note that models explicitly separating different sources of overdispersion exist [30], but their identifiability usually hinges on certain quantities being known from external sources (e.g., reporting probabilities).

In the examples of influenza in US military personnel and (to a lesser degree) COVID-19 in Austria, the model appeared to be mis-specified due to weekday patterns. These may inflate the estimated dispersion parameters and standard errors. More generally, estimating the dispersion parameter along with R from short time windows may lead to instabilities. For negative binomial regression, it is known that small-sample biases can occur [49], with a tendency to underestimate standard errors. Bias-corrected estimators exist and could be used instead of our ML estimators, but we do not expect this to have an impact on our conclusions. Finally, many other layers of complexity like unobserved initial generations in an outbreak [4] and changes in testing volumes [13] could be addressed. However, we consider these outside the scope of the present article.

A promising regression-based approach replacing sliding window approach by splines has recently been proposed by Nouvellet [10], and can remedy some of the aforementioned weaknesses. It estimates the temporal variation in R flexibly, potentially accounting for covariates like weekday effects. Unfortunately, the new framework currently only supports the Poisson distribution, and is thus likewise affected by the pitfalls we described. Extensions, however, could easily be implemented, potentially even allowing for time-varying dispersion parameters via the gamlss framework [43].

## Supporting information

10.1017/S0950268826101605.sm001 Němcová et al. supplementary material Němcová et al. supplementary material

## Supplementary material

The supplementary material for this article can be found at http://doi.org/10.1017/S0950268826101605.

## Reproducibility

The repository https://github.com/barbora-sobolova/epiestim_overdispesion/ contains code and data to reproduce our analyses.

## Figure 1 Long description

There are four horizontal rows, each representing a different parameter scenario, labeled on the left with *R*_t_, ξ, initialization, mean of the serial interval distribution, and standard deviation of the serial interval. In each row, the leftmost panel is a line graph with x-axis labeled Day and y-axis labeled Cases, showing 1,000 simulated incidence trajectories generated by the NegBin-L model. The x-axis is divided into Initialization, Burn-in period, and 14-day window segments, with a 7-day window indicated. The right two panels in each row are line graphs of empirical coverage versus nominal coverage, with y-axis labeled Empirical coverage and x-axis labeled Nominal coverage. The middle panel uses a 7-day window, the rightmost uses a 14-day window. Each coverage plot contains four colored lines for Poisson, quasi-Poisson, NegBin-L, and NegBin-Q models, with a dashed black line for theoretical Poisson coverage. Coverage lines generally follow the diagonal, except for the Poisson model, which indicates undercoverage. The legend on the far right identifies model colors and the dashed line. All axes, labels, and legends are precisely rendered as described.

Navigate back to Figure 1.

## Figure 2 Long description

The matrix consists of four rows and three columns. The columns, from left to right, represent influenza in USA active military personnel (2009–2010), COVID-19 in Austria (2021–2022), and Ebola in Guinea (2014–2015). The rows, from top to bottom, display: Row 1: Incidence curves with y-axis labeled ‘Cases’ and x-axis labeled ‘Date.’ Influenza and COVID-19 show daily cases, Ebola shows weekly cases. Row 2: Poisson model estimates of R, with y-axis labeled ‘R’ and x-axis labeled ‘Date.’ Row 3: quasi-Poisson model estimates of R, with shaded uncertainty bands. Row 4: Comparison of NegBin-L and NegBin-Q models, with overlapping shaded regions in blue and pink representing the confidence intervals. The legend at the bottom right identifies model colors: NegBin-L (blue), NegBin-Q (pink), Poiss (teal), Q-Poiss (yellow). All estimates are aligned to the last day of their estimation window. The COVID-19 incidence panel shows a sharp rise in cases, while influenza and Ebola show multiple peaks. R estimates show wider uncertainty for Ebola and influenza in the Quasi-Poisson and NegBin models.

Navigate back to Figure 2.

## Table 1 Long description

The table has seven columns: Reference, Disease, Location, Year, Resolution, Serial interval mean with standard deviation, and Estimation window width T. The first row lists pandemic influenza in US military personnel from 2009 to 2010, with daily resolution, serial interval mean 3.6 days with standard deviation 1.6, and estimation window width 7 days. The second row lists COVID-19 in Austria from 2021 to 2022, daily resolution, serial interval mean 3.37 days with standard deviation 1.83, and estimation window width 13 days. The third row lists Ebola in Guinea from 2014 to 2015, weekly resolution, serial interval mean 2.19 weeks with standard deviation 1.33, and estimation window width 4 weeks. All references are cited by number.

Navigate back to Table 1.

## Table 2 Long description

Starting from the top row, the table lists diseases: Pandemic influenza, COVID-19, and Ebola. For influenza, Poisson mean AIC is 7,517.84, NegBin-L mean AIC is 122.74, NegBin-Q mean AIC is 122.06. Both NegBin-L and NegBin-Q have lower AIC than Poisson in 100 percent of windows and 100 percent of likelihood ratio test rejections. For COVID-19, Poisson mean AIC is 11,926.48, NegBin-L mean AIC is 243.77, NegBin-Q mean AIC is 243.98. Both NegBin-L and NegBin-Q have lower AIC than Poisson in 100 percent of windows and 100 percent of likelihood ratio test rejections. For Ebola, Poisson mean AIC is 44.73, NegBin-L mean AIC is 35.44, NegBin-Q mean AIC is 35.53. NegBin-L has lower AIC than Poisson in 79.03 percent of windows and 75.81 percent of likelihood ratio test rejections; NegBin-Q has lower AIC than Poisson in 77.42 percent of windows and 75.81 percent of likelihood ratio test rejections.

Navigate back to Table 2.
