# Supplementary material for: Unjustified Poisson assumptions lead to overconfident estimates of the effective reproductive number
Source: Epidemiol Infect. 2026 May 25;154:e79. doi: 10.1017/S0950268826101605 (PMC13279973; doi:10.1017/S0950268826101605)
Supplement: Němcová et al. supplementary material [file S0950268826101605sup001.pdf]

Supplementary material for Němcová et al.:Unjustified Poisson assumptions lead to overconfident estimates of the effective reproductive number

* Correspondence to: Barbora Němcová (barbora.nemcova@kit.edu)

A Intuitive illustration of maximum likelihood estimation

To strengthen intuition, we provide a graphical illustration of likelihood inference under different models. The top panel of Figure S1 shows a 7-day window (3–9 January 2010) taken from the US influenza data discussed in Section 3.2. The incidence is overlaid with Λ_t from equation (1). The remaining panels show the log-likelihood contributions of the seven individual observations (grey) and the overall log-likelihood functions (black) for the Poisson and negative binomial models, all on a relative scale. For the negative binomial versions, the dispersion parameters are fixed at the maximum likelihood estimates.

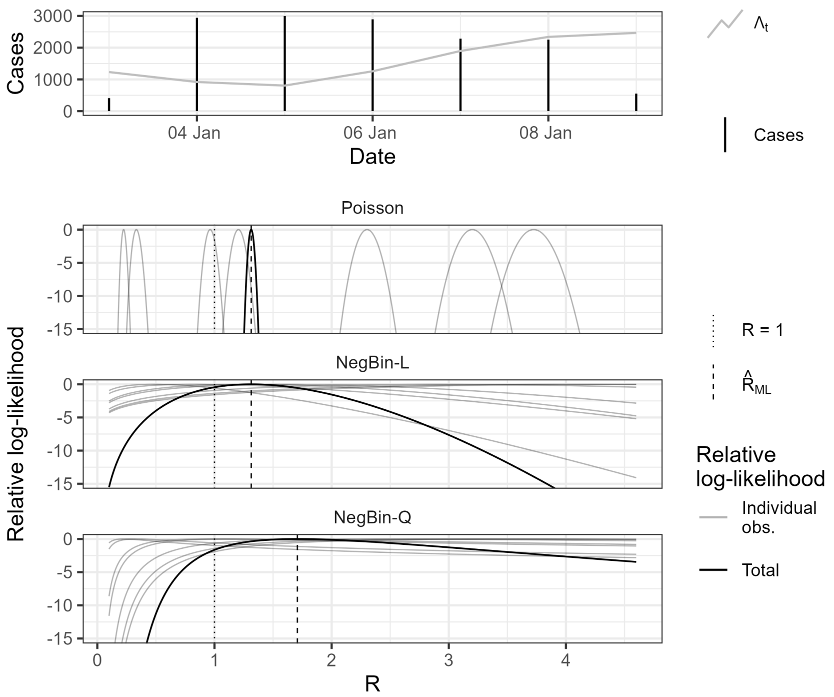

Figure S1: Illustration of maximum likelihood estimation in the Poisson, NegBin-L and NegBin-Q models. In the three bottom panels, light grey lines show the individual likelihood contributions, while black lines show the overall likelihood functions (i.e., the sums of the respective contributions). All of these are shown on a relative scale such that their peak value is zero. Maximum likelihood estimates are highlighted by vertical dashed lines, while the threshold value $R = 1.0$ is marked by a dotted line.

Due to the equidispersion assumption and high observed counts, the Poisson log-likelihood contribution functions are narrow, and the overall log-likelihood function exhibits a sharp peak. The curves for the negative binomial models are flatter as the model adapts to the higher degree of dispersion. Since $\widehat{\text{se}}(\hat{R})$ reflects the inverse curvature of the log-likelihood function at its peak (see Supplement B.1.1), it is clear that the Poisson model issues much more confident estimates. Notably, the Poisson likelihood clearly favours a value of $R > 1$, while for the negative binomial models, the relative likelihood is rather high for $R = 1$.

We moreover notice a shift in the point estimates from different models (dashed vertical lines). While they are almost identical for the Poisson and NegBin-L models ($\hat{R}_{\text{Po}} \approx \hat{R}_{\text{NBL}} \approx 1.31$), we obtain a higher value for NegBin-Q ($\hat{R}_{\text{NBQ}} \approx 1.71$). This is a result of the particular alignment of Λ_t and X_t, caused by weekday effects, and the different weights the $T = 7$ observations receive in the two schemes. To understand this, note that both estimators are weighted averages of the daily ratios $X_t/\Lambda_t, t = 1, \dots, T$. For the NegBin-Q model, this average is unweighted as in equation (8). For the Poisson model, equation (2) can be written as

$$\hat{R}_{\text{Po}} \approx \frac{1}{T} \times \sum_{t=1}^{T} \frac{\Lambda_t}{\sum_{t=1}^{T} \Lambda_t} \times \frac{X_t}{\Lambda_t},$$

and also applies in good approximation for the NegBin-L model [1, p. 199]. Here, the ratios X_t/Λ_t thus enter with weights proportional to Λ_t. As seen in the top panel of Figure S1, the highest values of X_t occur for the lowest values of Λ_t. These differences in weighting thus explain why the NegBin-Q estimate is considerably higher than its counterparts from the Poisson and NegBin-L models.

B Mathematical details

B.1 Details on the Poisson model, equation (1) in the main text

B.1.1 Maximum likelihood estimation (equations (2) and (3) in the main text)

Here we derive the formulas (2) and (3) using standard maximum likelihood theory. In the following we denote the vector of observed values in the estimation window by $\mathbf{X} = (X_1, \dots, X_T)$ and substitute $\mu_t = R\Lambda_t$. Due to the D-th order Markov structure of the Poisson renewal equation model (1), the log-likelihood is given by a sum of T Poisson log-likelihoods,

$$l(R; \mathbf{X}) = \sum_{t=1}^{T} \log\left\{\frac{(R\Lambda_t)^{X_t}}{X_t!} \times \exp(-R\Lambda_t)\right\} = \sum_{t=1}^{T} \left\{X_t \log(R) - R\Lambda_t + X_t \log(\Lambda_t) - \log(X_t!)\right\}. \quad \text{(S1)}$$

Taking the derivative with respect to R we obtain the score function

$$\frac{\partial l(R; \mathbf{X})}{\partial R} = \frac{\partial}{\partial R}\left\{\sum_{t=1}^{T} \left\{X_t \log(R) - R\Lambda_t + X_t \log(\Lambda_t) - \log(X_t!)\right\}\right\} = \sum_{t=1}^{T}\left\{\frac{X_t}{R} - \Lambda_t\right\}.$$

Next we set the score function equal to zero and solve for R,

$$\sum_{t=1}^{T}\left\{\frac{X_t}{R} - \Lambda_t\right\} \equiv 0. \quad \Rightarrow \quad \hat{R}_{\text{Po}} = \frac{\sum_{t=1}^{T} X_t}{\sum_{t=1}^{T} \Lambda_t},$$

which results in the maximum likelihood estimator. In order to find an estimate of its variance, we compute the observed Fisher information, which involves taking the second derivative of the log-likelihood function,

$$\mathcal{J}(R) = -\frac{\partial^2 l(R; \mathbf{X})}{\partial R^2} = \sum_{t=1}^{T} \frac{X_t}{R^2}. \tag{S2}$$

We then obtain the formula of the standard error of $\hat{R}_{\text{Po}}$ by inverting the observed Fisher information at the maximum likelihood estimate and taking the square root,

$$\widehat{\text{se}}(\hat{R}_{\text{Po}}) = \sqrt{\left(\sum_{t=1}^{T} \frac{X_t}{\hat{R}_{\text{Po}}^2}\right)^{-1}} = \frac{\hat{R}_{\text{Po}}}{\sqrt{\sum_{t=1}^{T} X_t}}.$$

We note that unlike in a classic generalized linear regression model (GLM), the "covariate" Λ_t is itself random, and all $\Lambda_t, X_t, t = 1, \ldots, T$ are dependent. This invalidates some of the basic assumptions underlying maximum likelihood theory for GLMs. Due to the Markov structure of the process (1), however, the log-likelihood function (S1) and the observed Fisher information (S2) are nonetheless correct, and the `glm` implementation in R can be used to evaluate them. It is non-trivial to show that maximum likelihood estimators preserve their usual properties in stochastic process models like (1), as relevant mixing properties need to be established. This has been done in related stationary models from the INGARCH family (see e.g., [2]), but in practice maximum likelihood estimators and associated standard errors are widely used and have been found to be reliable even when their properties have not been formally demonstrated (see e.g., [3]). For model (1), an additional challenge arises from the fact that the process is non-stationary and potentially explosive. Mixing properties in related settings have been explored by [4], but we consider these aspects outside the scope of the present applied article.

B.1.2 Comparison to Bayesian estimation

In the `EpiEstim` package, Bayesian estimation of R in the model (1) is based on a conjugate gamma prior distribution. Specifying the prior distribution as

$$R \sim \text{Gamma}(\text{shape} = a, \text{scale} = b),$$

the posterior distribution given the data $\mathbf{X} = (X_1, \ldots, X_T)$ is likewise a gamma distribution. Specifically, we obtain [5, Supplementary Material, page 3]

$$R \mid \mathbf{X} \sim \text{Gamma}\left(\text{shape} = a + \sum_{t=1}^{T} X_t, \text{scale} = \frac{1}{\frac{1}{b} + \sum_{t=1}^{T} \Lambda_t}\right).$$

The first two posterior moments of R are consequently given by

$$\mathbb{E}(R \mid \mathbf{X}) = \frac{a + \sum_{t=1}^{T} X_t}{\frac{1}{b} + \sum_{t=1}^{T} \Lambda_t},$$

$$\text{sd}(R \mid \mathbf{X}) = \frac{\mathbb{E}(R \mid \mathbf{X})^2}{\sqrt{a + \sum_{t=1}^{T} X_t}}.$$

This strongly resembles the maximum likelihood estimator $\hat{R}_{\text{Po}}$ from (2) and its estimated standard deviation given in equation (3). Indeed, the Bayesian scheme can be seen as a regularized maximum likelihood estimate resulting from the addition of a pseudo observation with $\Lambda_0 = 1/b$ and $X_0 = a$. The default values in `EpiEstim` are $a = 1, b = 1/5$, meaning that for moderately high incidence values they have little influence on the result.

B.2 Details on the quasi-Poisson model

B.2.1 Estimation

The most common way to estimate the dispersion parameter $\hat{\phi}$, implemented in the `R` function `glm(..., family = quasipoisson)`, is

$$\hat{\phi} = \frac{1}{T-1} \sum_{t=1}^{T} \frac{(X_t - \hat{R}_{\text{Po}} \Lambda_t)^2}{\hat{R}_{\text{Po}} \Lambda_t}. \tag{S3}$$

This is simply the empirical variance of the Pearson residuals in the Poisson model. The standard errors in expression (3) are then corrected to

$$\widehat{\text{se}}(\hat{R}_{\text{Po}}) = \sqrt{\hat{\phi}} \times \frac{\hat{R}^2_{\text{Po}}}{\sqrt{\sum_{t=1}^{T} X_t}}. \tag{S4}$$

B.2.2 Implications for confidence intervals

For ϕ fixed, the $(1-\gamma)$ Wald confidence interval of the quasi-Poisson model becomes

$$\hat{R}_{\text{Po}} \pm \Phi^{-1}\left(1 - \frac{\gamma}{2}\right) \times \underbrace{\sqrt{\phi} \times \frac{\hat{R}^2_{\text{Po}}}{\sqrt{\sum_{t=1}^{T} X_t}}}_{u}, \tag{S5}$$

where $\Phi(\cdot)$ denotes the cumulative probability function of the standard normal distribution and $\Phi^{-1}(\cdot)$ its quantile function.

If the assumptions of the quasi-Poisson regression are fulfilled, we can use this to derive the expected coverage level of uncorrected Poisson-based confidence intervals. To this end, note that the quasi-Poisson interval at level $(1-\gamma)$ is constructed such that

$$\Pr\left(R \in \left[\hat{R}_{\text{Po}} - \Phi^{-1}\left(1 - \frac{\gamma}{2}\right) \times u, \hat{R}_{\text{Po}} + \Phi^{-1}\left(1 - \frac{\gamma}{2}\right) \times u\right]\right) = 1 - \gamma. \tag{S6}$$

Now setting

$$\frac{\gamma}{2} = \Phi\left[\Phi^{-1}\left(\frac{\alpha}{2}\right) \times \frac{1}{\sqrt{\phi}}\right], \tag{S7}$$

it is easy to show that the $(1-\gamma)$ confidence interval of the quasi-Poisson model and the $(1-\alpha)$ interval of the Poisson are identical. This is because the relationship between the $(1-\gamma/2)$ and $(1-\alpha/2)$ quantiles of the standard normal distribution is as follows

$$\begin{aligned} \Phi^{-1}\left(1 - \frac{\gamma}{2}\right) &= \Phi^{-1}\left(1 - \Phi\left[\Phi^{-1}\left(\frac{\alpha}{2}\right) \times \frac{1}{\sqrt{\phi}}\right]\right), \\ &= \Phi^{-1}\left(\Phi\left[-\Phi^{-1}\left(\frac{\alpha}{2}\right) \times \frac{1}{\sqrt{\phi}}\right]\right), \\ &= -\Phi^{-1}\left(\frac{\alpha}{2}\right) \times \frac{1}{\sqrt{\phi}}, \\ &= \Phi^{-1}\left(1 - \frac{\alpha}{2}\right) \times \frac{1}{\sqrt{\phi}}. \end{aligned} \tag{S8}$$

Substituting for $\Phi^{-1}(1-\gamma/2)$ in (S5) we get

$$\hat{R}_{\text{Po}} \pm \Phi^{-1}\left(1 - \frac{\alpha}{2}\right) \times \frac{u}{\sqrt{\phi}} = \hat{R}_{\text{Po}} \pm \Phi^{-1}\left(1 - \frac{\alpha}{2}\right) \times \underbrace{\frac{\hat{R}^2_{\text{Po}}}{\sqrt{\sum_{t=1}^{T} X_t}}}_{\substack{\widehat{\text{se}} \text{ of the Poisson} \\ \text{model}}}, \tag{S9}$$

which is the formula of the $(1-\alpha)$ Wald confidence interval of the Poisson model. Now, constructing this $(1-\alpha)$ Poisson confidence interval, while the quasi-Poisson model holds, corresponds to plugging (S8) into (S6), which yields the expected coverage

$$\Pr\left(R \in \left[\hat{R}_{\text{Po}} - \Phi^{-1}\left(1-\frac{\alpha}{2}\right) \times \underbrace{\frac{u}{\sqrt{\phi}}}_{\substack{\widehat{\text{se}} \text{ of the} \\ \text{Poisson model}}}, \hat{R}_{\text{Po}} + \Phi^{-1}\left(1-\frac{\alpha}{2}\right) \times \underbrace{\frac{u}{\sqrt{\phi}}}_{\substack{\widehat{\text{se}} \text{ of the} \\ \text{Poisson model}}}\right]\right) = 1 - 2 \times \Phi\left[\Phi^{-1}\left(\frac{\alpha}{2}\right) \times \frac{1}{\sqrt{\phi}}\right]$$

of the Poisson-based confidence interval.

B.3 Details on the NegBin-Q model, equation (7) in the main text

B.3.1 Maximum likelihood estimation, equations (8) and (9) in the main text

The negative binomial distribution with mean μ and dispersion parameter ψ has probability mass function

$$f(x) = \frac{\Gamma(\psi + x)}{x!\Gamma(\psi)} \left(\frac{\psi}{\psi + \mu}\right)^{\psi} \left(\frac{\mu}{\psi + \mu}\right)^{x}.$$

Following the same logic as in Supplement B.1.1, the log-likelihood function of the NegBin-Q model (7) is

$$\begin{aligned} l(R; \mathbf{X}) &= \sum_{t=1}^{T} \log[f(X_t | R, \Lambda_t)] \\ &= \sum_{t=1}^{T} \Big\{ \log\left[\Gamma(\psi + X_t)\right] - \log\left[X_t!\Gamma(\psi)\right] + \psi \times \log(\psi) \\ &\qquad - (X_t + \psi) \times \log(\psi + R\Lambda_t) + X_t \log(R\Lambda_t) \Big\}. \end{aligned}$$

Now we differentiate with respect to R to obtain the score equation

$$\begin{aligned} \frac{\partial l(R; \mathbf{X})}{\partial R} &= \sum_{t=1}^{T} \left\{ (X_t + \psi) \times \frac{-\Lambda_t}{R\Lambda_t + \psi} + \frac{X_t}{R} \right\} \\ &= \ldots = \\ &= \sum_{t=1}^{T} \frac{\psi(X_t - R\Lambda_t)}{R(R\Lambda_t + \psi)} \equiv 0, \end{aligned}$$

which is equivalent to

$$\sum_{t=1}^{T} \frac{X_t - R\Lambda_t}{R\Lambda_t + \psi} \equiv 0. \tag{S10}$$

We now assume that there is substantial overdispersion, which we formalize as

$$\psi \ll \mu_t = R\Lambda_t \quad \text{for} \quad t = 1, \ldots, T,$$

implying that

$$\sigma_t^2 \approx \frac{\mu_t^2}{\psi}.$$

Equation (S10) then simplifies to the estimator from equation (8) as

$$\begin{aligned} & \sum_{t=1}^{T} \left(\frac{X_t}{R\Lambda_t} - 1 \right) \approx 0 \\ \Leftrightarrow \quad & \frac{1}{R} \times \sum_{t=1}^{T} \frac{X_t}{\Lambda_t} \approx T \\ \Rightarrow \quad & \hat{R}_{\text{NBQ}} \approx \frac{1}{T} \times \sum_{t=1}^{T} \frac{X_t}{\Lambda_t}. \end{aligned} \tag{S11}$$

Next we again compute the observed Fisher information with respect to R,

$$\begin{aligned} \mathcal{J}(R) = -\left(\frac{\partial^2 l(R; \mathbf{X})}{\partial R^2} \right) &= -\frac{\partial}{\partial R} \left(\sum_{t=1}^{T} \frac{\psi(X_t - R\Lambda_t)}{R(R\Lambda_t + \psi)} \right) \\ &= \sum_{t=1}^{T} \frac{\psi \Lambda_t R(R\Lambda_t + \psi) + \psi(X_t - R\Lambda_t)(2R\Lambda_t + \psi)}{R^2(R\Lambda_t + \psi)^2} \\ &= \ldots = \sum_{t=1}^{T} \frac{\psi X_t(2R\Lambda_t + \psi) - \psi R^2 \Lambda_t^2}{R^2(R\Lambda_t + \psi)^2}, \end{aligned}$$

Now again assuming that $\psi \ll \mu_t = R\Lambda_t$ this simplifies to

$$\begin{aligned} \mathcal{J}(R) &\approx \sum_{t=1}^{T} \frac{2\psi X_t R\Lambda_t - \psi R^2 \Lambda_t^2}{R^4 \Lambda_t^2}, \\ &= \frac{\psi}{R^2} \times \left[\frac{2}{R} \times \sum_{t=1}^{T} \frac{X_t}{\Lambda_t} \quad + \quad \sum_{t=1}^{T} -1 \right] \end{aligned}$$

Now evaluating this at the (approximate) maximum likelihood estimator (S11) we obtain

$$\begin{aligned} \mathcal{J}(\hat{R}_{\text{NBQ}}) &= \frac{\psi}{\hat{R}_{\text{NBQ}}^2} \times \left[\underbrace{\frac{2}{\sum_{t=1}^{T} \frac{X_t}{\Lambda_t}} \times \sum_{t=1}^{T} \frac{X_t}{\Lambda_t}}_{=2T} \quad + \quad -T \right] \\ &= \frac{\psi T}{\hat{R}_{\text{NBQ}}^2}. \end{aligned}$$

As before, the standard error of $\hat{R}_{\text{NBQ}}$ then results as

$$\widehat{\text{se}}(\hat{R}_{\text{NBQ}}) = \sqrt{\frac{1}{\mathcal{J}(\hat{R}_{\text{NBQ}})}} = \hat{R}_{\text{NBQ}} \times \sqrt{\frac{1}{\psi T}}.$$

B.3.2 Estimation of the dispersion parameter ψ

The dispersion parameter ψ can be estimated as

$$\hat{\psi} = \left(\frac{1}{T-1} \sum_{t=1}^{T} \frac{(X_t - \hat{R}_{\text{NBQ}}\Lambda_t)^2}{(\hat{R}_{\text{NBQ}}\Lambda_t)^2} \right)^{-1}, \tag{S12}$$

which resembles the inverse of equation (S3), but is different due to the square $(\hat{R}_{\text{NBQ}}\Lambda_t)^2$. This estimator, which to our knowledge has not been proposed previously, is motivated by the fact that under model (7) we have

$$\sigma_t^2 = \text{Var}(X_t \mid \mu_t) = \mu_t + \frac{\mu_t^2}{\psi}.$$

Under our additional assumption $\psi \ll \mu_t$ this simplifies to

$$\begin{aligned} &\text{Var}(X_t \mid \mu_t) = \frac{\mu_t^2}{\psi} \\ \Leftrightarrow\ &\text{Var}\left(\underbrace{\frac{X_t - \mu_t}{\mu_t}}_{\text{denote this by } r_t} \;\middle|\; \mu_t \right) = \frac{1}{\psi}. \end{aligned}$$

Now note that we moreover have

$$\mathbb{E}\left(r_t \mid \mu_t\right) = 0.$$

Due to the Markov property of $\{X_t\}$, the $r_1, \ldots, r_T$ are hence independent random variables with mean zero and identical variance $1/\psi$. The inverse of the empirical variance (S12) of $r_1, \ldots, r_T$ is thus a natural estimator of ψ.

C Details on the simulation study

C.1 Empirical distribution of the $\hat{R}$ estimates

Supplementary Figure S2 shows the empirical distribution of the $\hat{R}$ estimates across the different scenarios and 1000 simulation runs each. The point estimates follow similar distributions across all four models and scenarios. In contrast, clear differences can be spotted in the distribution of estimated standard errors. The Poisson model produces consistently low estimated standard errors, whereas models accounting for overdispersion yield a broader range of higher values.

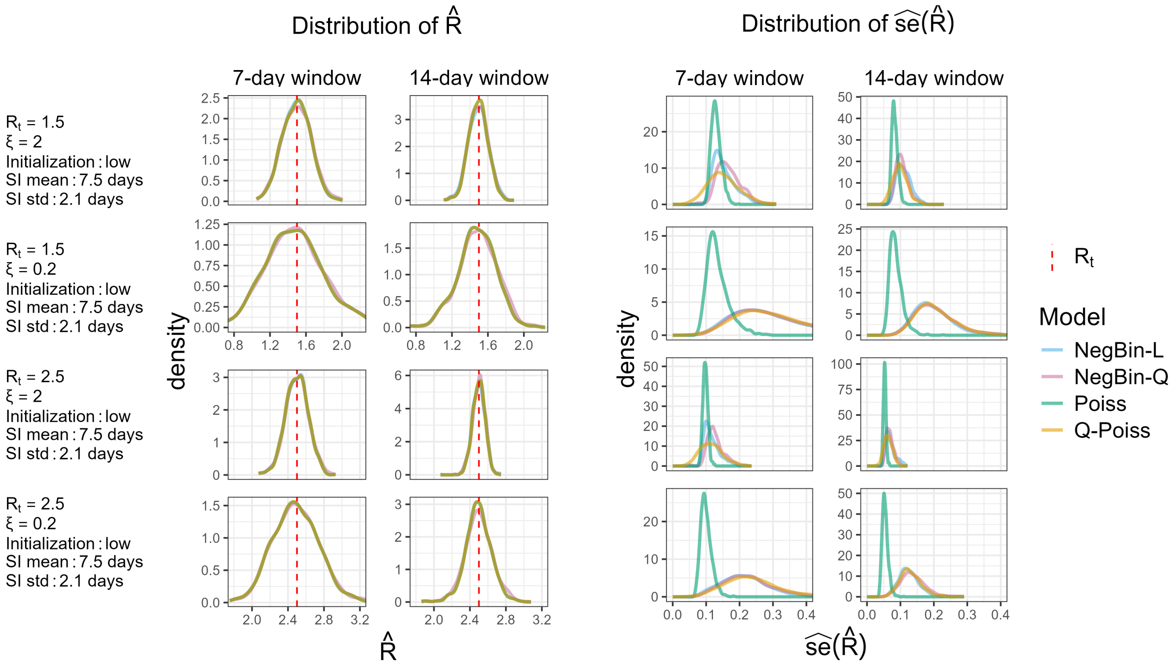

Figure S2: Empirical distribution of the $\hat{R}$ estimates (two left columns) and their standard errors (two right columns) under the NegBin-L distributional assumptions, low initialization values and a serial interval typical for RSV. Different parameter combinations are specified on the left margin of the figure. The red dashed line shows the true value of the effective reproductive number R.

C.2 Supplementary simulation scenarios

In this section, we provide additional simulation scenarios to demonstrate that the overconfidence of Poisson confidence intervals for estimates of effective reproductive number R persists across different data generating mechanisms and parameter values. In Section 3.1 of the main manuscript, trajectories were generated using the NegBin-L model, combining two values of R, two levels of overdispersion, a single set of initial values and one particular serial interval distribution. In the supplementary scenarios we keep the combination of the two R values and the two dispersion levels, but we additionally vary the initial values, the serial interval and the distribution of the reported incidence. Additionally, we include several scenarios, where R is not constant over time. Supplementary Table S1 provides a summary of such scenario blocks and corresponding figures.

Table S1: Summary of simulation scenario blocks and

Data generating process	Serial interval mean (sd)	Initial values	R-value	Figure	Section
NegBin-L	7.5 (2.1)	low	constant	1, S2 and S17	3.1
NegBin-L	7.5 (2.1)	high	constant	S3, S4, S17	C.2.1
NegBin-L	13.7 (1.5)	low	constant	S5, S6 and S17	C.2.2
NegBin-L	3.7 (1.1)	low	constant	S7, S8 and S17	C.2.2
NegBin-L	7.5 (2.1)	low and high	time-varying	S10	C.2.3
NegBin-Q	7.5 (2.1)	low and high	constant	S11, S12 and S18	C.2.4
Poisson	7.5 (2.1)	low and high	constant	S13 and S14	C.2.5
Branching process	7.5 (2.1)	low	constant	S15 and S16	C.2.6

C.2.1 NegBin-L simulation scenarios with higher initial values

In this section, we show four simulation scenarios, which, compared to those presented in Section 3.1 of the main paper, use higher values to initialize the trajectories to show that the coverage of the overdispersed models remains good for higher overall incidence. We again generate 1000 trajectories from the NegBin-L renewal equation model. The model parameters remain the same as in the main manuscript, in particular $R \in \{1.5, 2.5\}$ and $\xi \in \{2, 0.2\}$ and we use a serial interval with mean 7.5 and standard error 2.1. However, the fixed initial values are: (113, 100, 106, 102, 87, 99, 103, 102, 103, 97, 98, 97, 107, 86), producing trajectories with overall higher magnitude. The results are very similar to the scenarios from the main manuscript. The empirical coverage of confidence intervals from the overdispersed models is close to the nominal levels. For scenarios with lower dispersion, the undercoverage of the Poisson model is moderate, while for more dispersed trajectories, the Poisson model heavily undercovers.

C.2.2 NegBin-L simulation scenarios with a different serial interval

In this section, we present a variation of the simulation study from Section 3.1 of the main manuscript, with different examples of the serial interval distribution. All other parameter values are kept the same as in the main manuscript, i.e. $R \in \{1.5, 2.5\}$ and $\xi \in \{2, 0.2\}$.

For scenarios shown in Figure S5 we use a serial interval distribution typical for measles [6], with mean 13.7 days and standard deviation of 1.5 days. We extended the burn-in period in these scenarios to 21 days, to make sure that it covers the whole support of the serial interval distribution and consequently we also extend the initialization sequence to (2, 7, 6, 4, 3, 7, 7, 6, 6, 6, 4, 9, 3, 7, 8, 5, 4, 5, 6, 3, 9). In Figure S5, the trajectories do not exhibit such a growth as in the scenarios in the main manuscript due to a very long time between infections. However, the pattern of the Poisson confidence intervals undercovering remains clear, as well as more accurate coverage achieved by the overdispersed models.

For scenarios presented in Figure S7, the mean serial interval is 3.7 days with the standard deviation of 2.1 days, which can be motivated by influenza [7], and we initialize the trajectory by the same fixed values as in the main manuscript: (2, 7, 6, 4, 3, 7, 7, 6, 6, 6, 4, 9, 3, 7). Compared to the scenarios from Section 3.1, the resulting trajectories are growing much faster, owing to the shorter serial interval. In these scenarios, too, our findings, that the overdispersed models exhibit better coverage than the Poisson model, remain consistent.

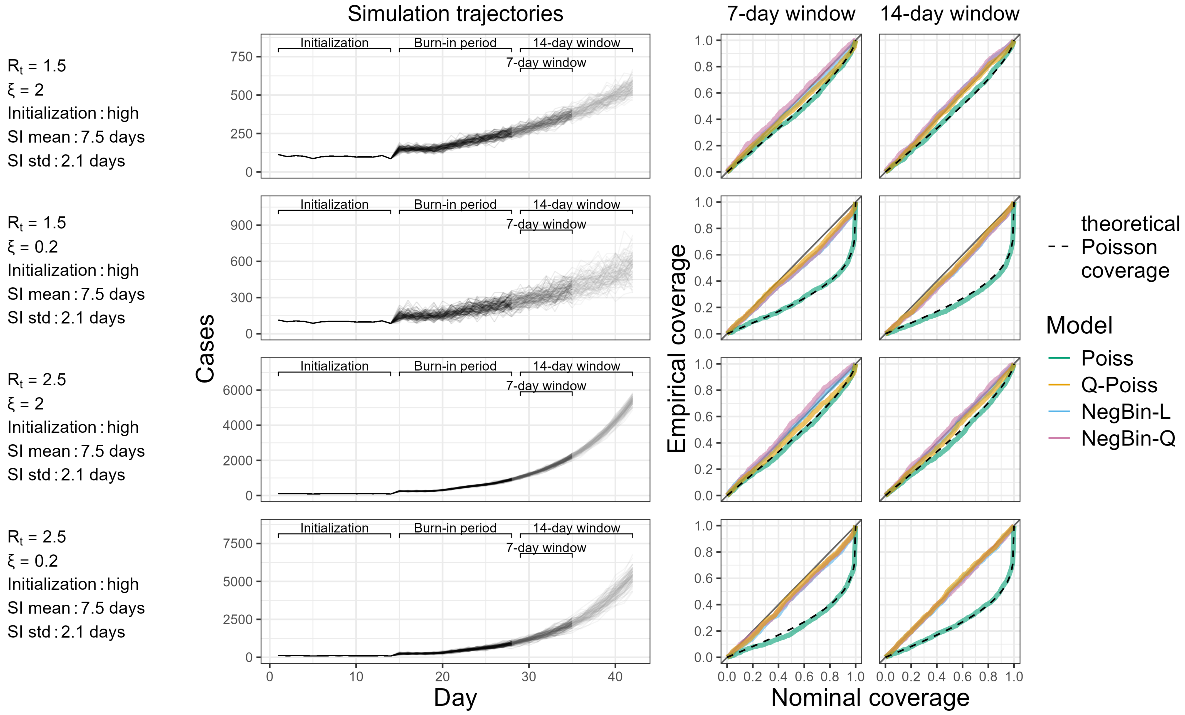

Figure S3: Simulation trajectories generated by the NegBin-L model and empirical coverage of the four models in scenarios initialized by high values, with constant R and a serial interval typical to RSV. The left panel shows 1000 incidence trajectories generated from the renewal equation with the NegBin-L distribution across four scenarios defined by different parameter combinations specified on the left margin of the figure. The right panels display empirical coverage of the true R for Poisson, quasi-Poisson and negative binomial models across nominal coverage levels, using 7-day (middle column) and 14-day (right column) estimation windows.

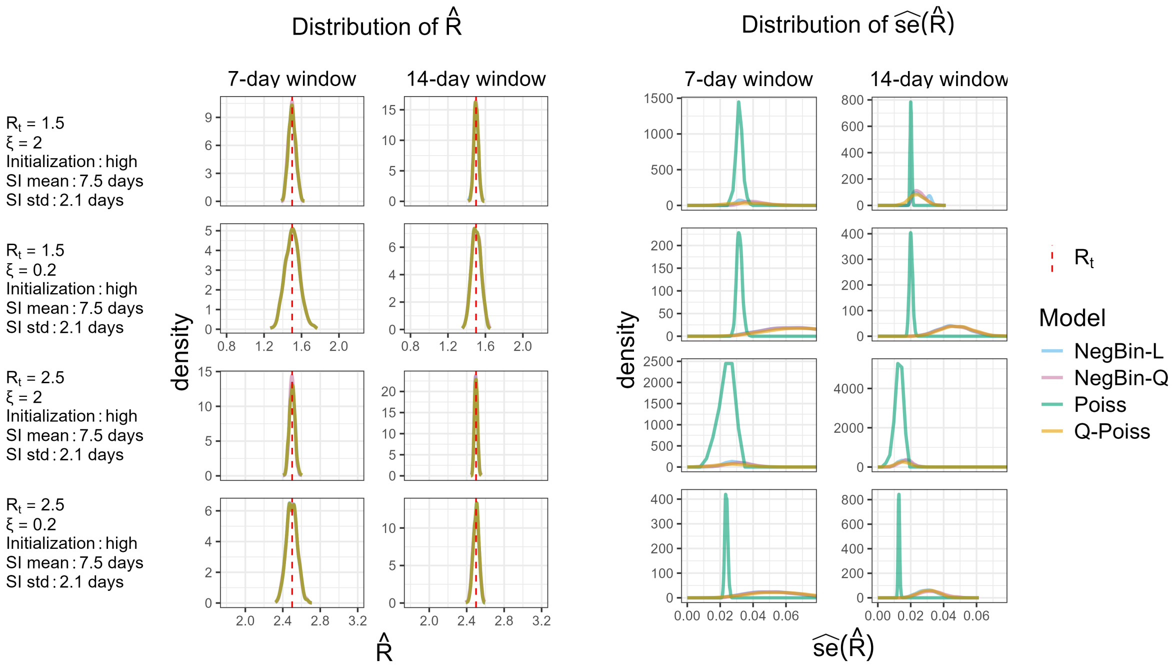

Figure S4: Empirical distribution of the $\hat{R}$ estimates (two left columns) and their standard errors (two right columns) under the NegBin-L distributional assumptions, high initialization values and a serial interval typical for RSV. Different parameter combinations are specified on the left margin of the figure. The red dashed line shows the true value of the effective reproductive number R.

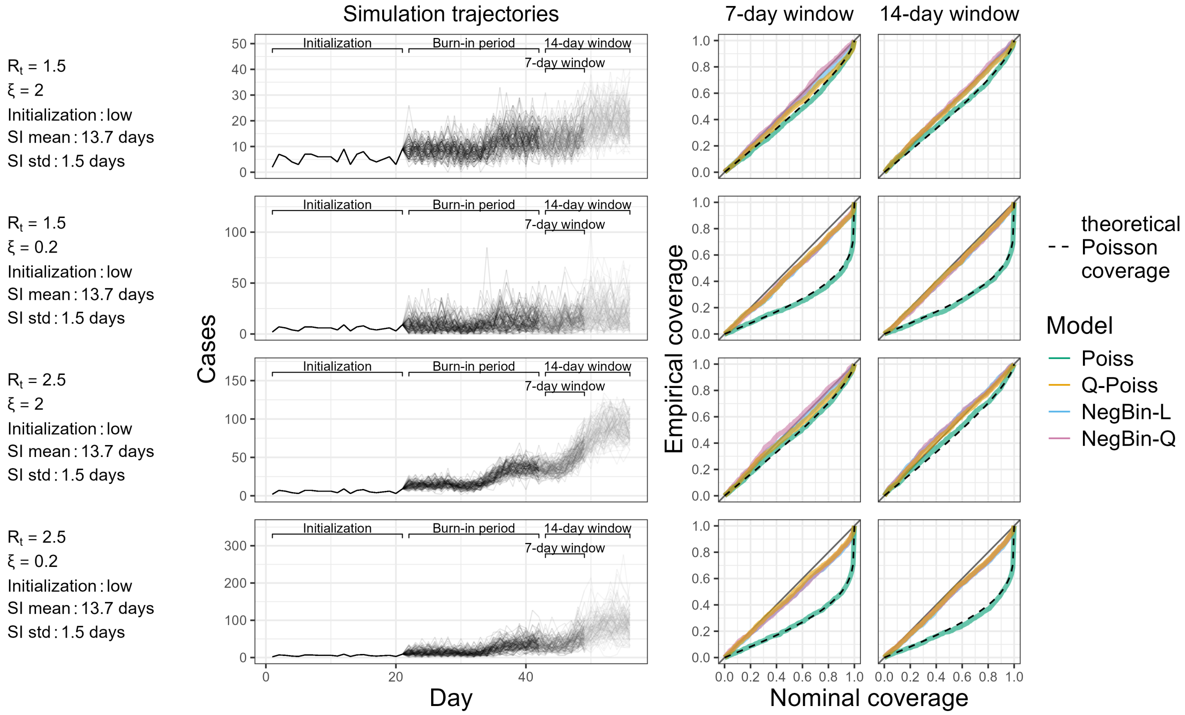

Figure S5: Simulation trajectories generated by the NegBin-L model and empirical coverage of the four models in scenarios initialized by low values, with constant R and a serial interval typical to measles. The left panel shows 1000 incidence trajectories generated from the renewal equation with the NegBin-L distribution across four scenarios defined by different parameter combinations specified on the left margin of the figure. The right panels display empirical coverage of the true R for Poisson, quasi-Poisson and negative binomial models across nominal coverage levels, using 7-day (middle column) and 14-day (right column) estimation windows.

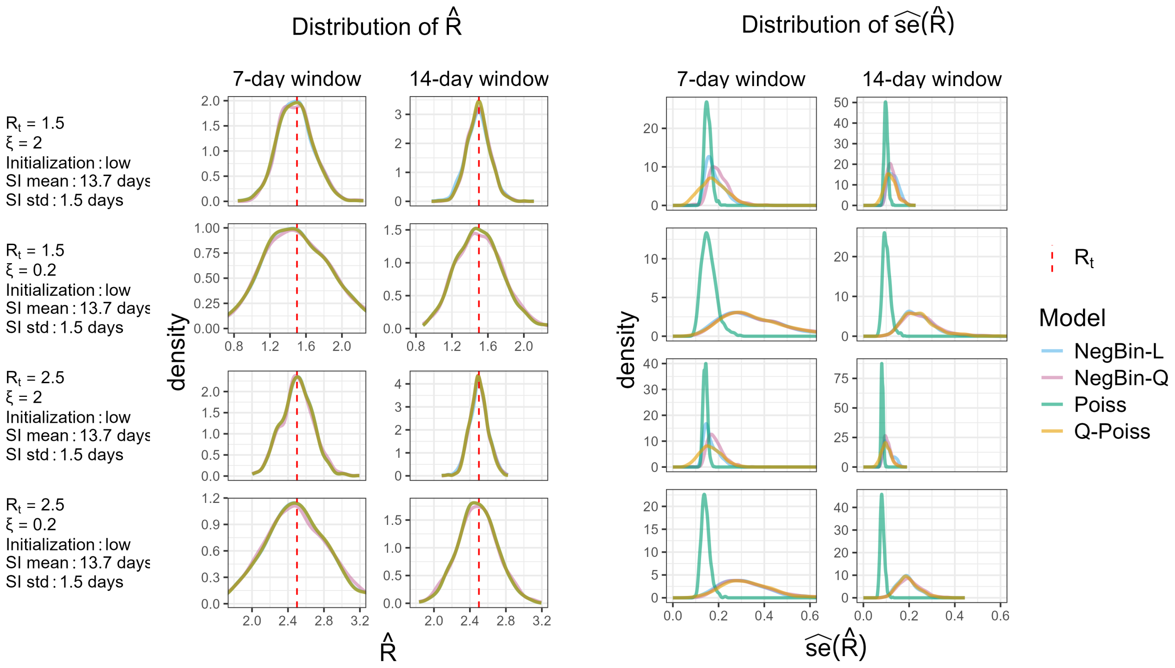

Figure S6: Empirical distribution of the $\hat{R}$ estimates (two left columns) and their standard errors (two right columns) under the NegBin-L distributional assumptions, low initialization values and a serial interval typical for measles. Different parameter combinations are specified on the left margin of the figure. The red dashed line shows the true value of the effective reproductive number R.

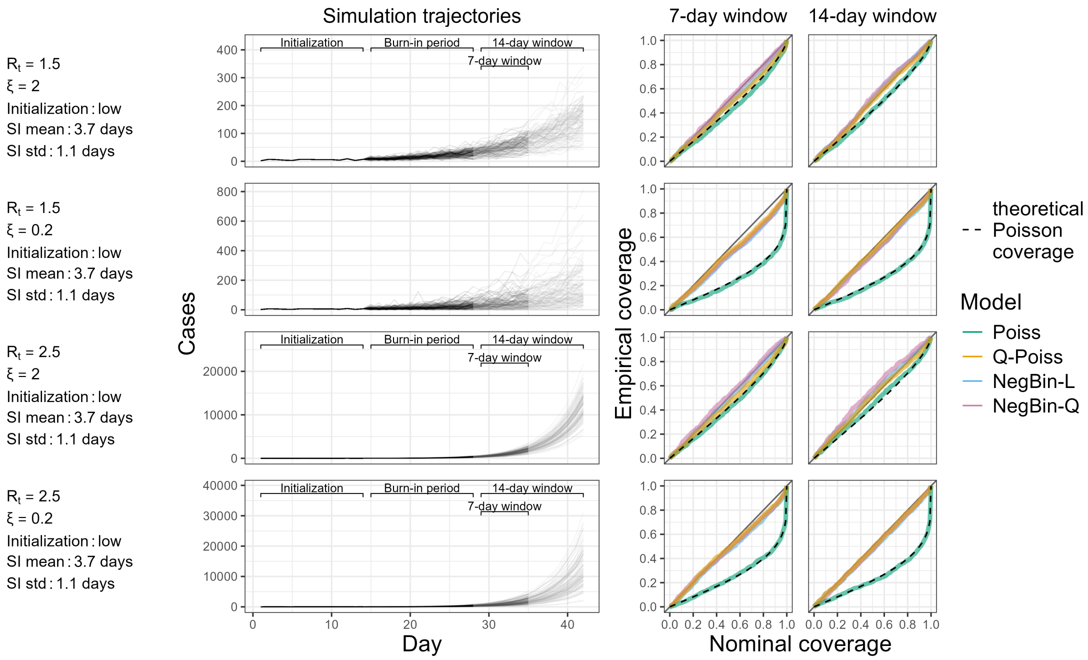

Figure S7: Simulation trajectories generated by the NegBin-L model and empirical coverage of the four models in scenarios initialized by low values, with constant R and a serial interval typical to influenza. The left panel shows 1000 incidence trajectories generated from the renewal equation with the NegBin-L distribution across four scenarios defined by different parameter combinations specified on the left margin of the figure. The right panels display empirical coverage of the true R for Poisson, quasi-Poisson and negative binomial models across nominal coverage levels, using 7-day (middle column) and 14-day (right column) estimation windows.

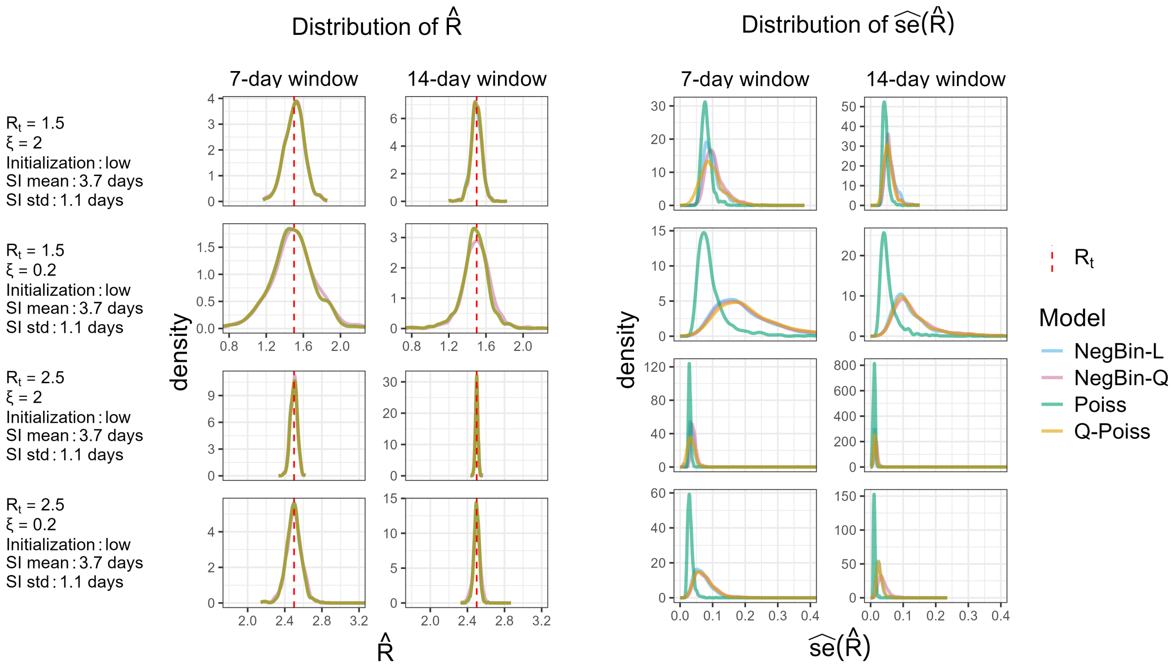

Figure S8: Empirical distribution of the $\hat{R}$ estimates (two left columns) and their standard errors (two right columns) under the NegBin-L distributional assumptions, low initialization values and a serial interval typical for influenza. Different parameter combinations are specified on the left margin of the figure. The red dashed line shows the true value of the effective reproductive number R.

C.2.3 NegBin-L simulation scenarios with time-varying R-values

To compare the renewal equation models in a more realistic setting, we conducted a variation of the simulation study, where the effective reproductive number R varies smoothly over time according to a cosine wave function:

$$R(t) = 0.11 \times \cos(2.5 \times \pi \times (t - 14)/28) + 1.5 \quad \text{(S13)}$$

The numerical values in Equation S13 are selected to create a curve that oscillates moderately around 1.5. In the 7-day estimation window, R_t increases from 1.45 to 1.6. The overall trajectory of $R(t)$ is shown in Figure S9. We note that we chose this moderate variation as stronger variation within a time window make it increasingly difficult to meaningfully summarize the seven values of R_t into a single "true" value that a confidence interval should cover.

We then simulated from the NegBin-L renewal equation model using the corresponding time-dependent values of R, two levels of dispersion $\xi \in \{2, 0.2\}$ and two different sets of initial values. The set of low initial values is the same as in Section 3.1 of the main manuscript, while for the initialization by higher values, we reused the sequence from Supplementary Section C.2.1.

Since the underlying value of R is no longer constant, there is no single true value, we can compare the estimates to. In order to nonetheless compute coverage fractions, we calculated either the arithmetic, or the geometric mean of the $R(t)$ trajectory within the corresponding estimation window and checked how well our models cover this particular value. In our example, the difference between the arithmetic and geometric mean is less than 0.001, which leads to almost identical coverage. For this reason, we present the coverage in Figure S10 only for the arithmetic mean.

Figure S10 shows similar patterns as Figure 1 and Supplementary Figure S3. Poison coverage is only slightly lower than the nominal levels when overdispersion is low. However, in scenarios with stronger overdispersion, the Poisson model again exhibits undercoverage, while the overdispersed models remain well-calibrated. This demonstrates, that the overconfidence of the Poisson model persists also in settings, where the value of R varies smoothly over time.

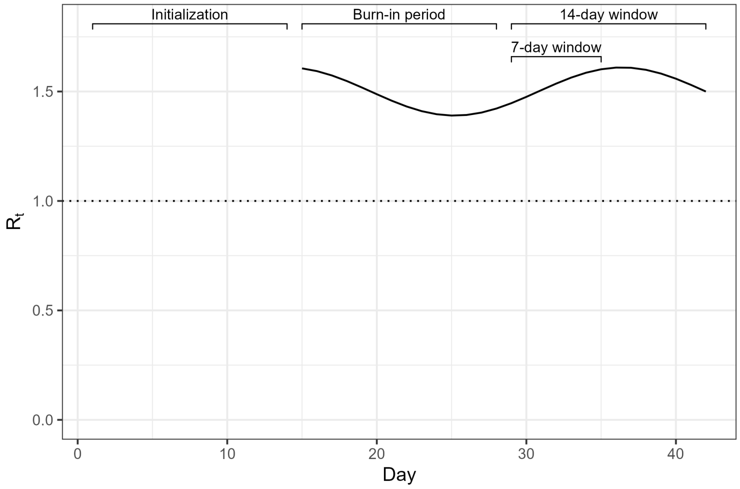

Figure S9: True values of R in time used to simulate incidence trajectories.

C.2.4 NegBin-Q simulation scenarios

We further simulated eight scenarios assuming the NegBin-Q distribution as the true data-generating process, instead of NegBin-L. We use the same values for the initialization of the trajectories as in Section 2.3 and Supplementary Section C.2.1. Similarly to the simulation in the main text, approximately 11% of simulation runs in low dispersion settings did not converge, requiring to revert to the Poisson model for these particular runs. The empirical coverage results, shown in Supplementary Figure S11, are overall

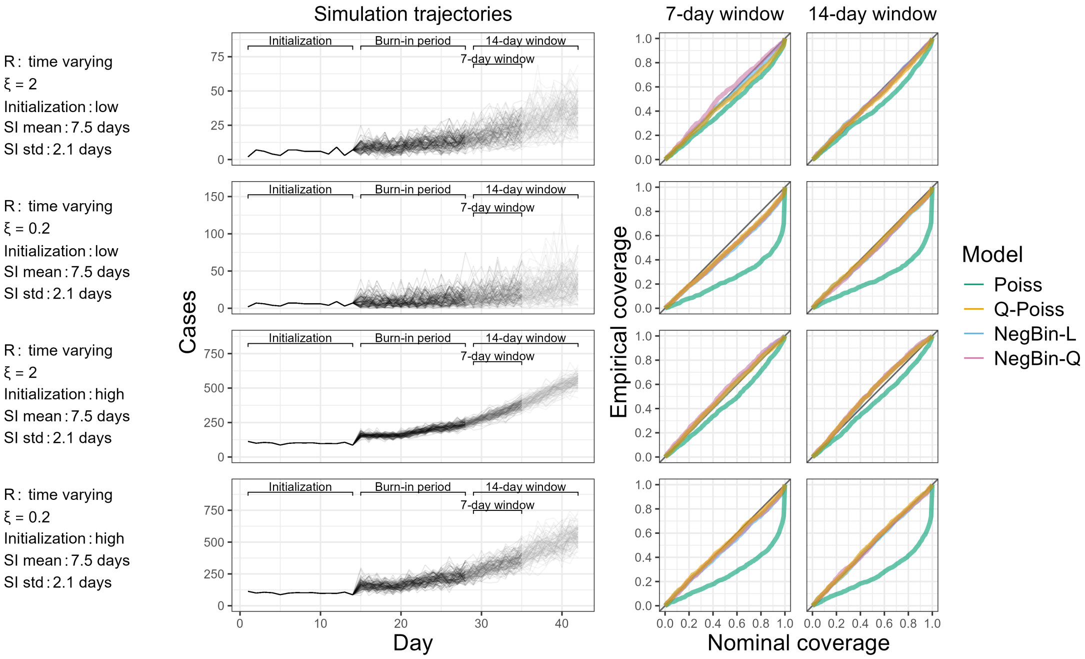

Figure S10: Simulation trajectories generated by the NegBin-L model and empirical coverage of the four models in scenarios initialized by low and high values, with time-varying R-values and a serial interval typical to RSV. The left panel shows 1000 incidence trajectories generated from the renewal equation with the NegBin-L distribution across four scenarios defined by different parameter combinations specified on the left margin of the figure. The right panels display empirical coverage of the arithmetic mean of the $R(t)$ trajectory across the 7-day (middle column) and 14-day (right column) estimation window for Poisson, quasi-Poisson and negative binomial models across nominal coverage levels.

similar to those from Figure 1 and Supplementary Figure S3. Again we see strong undercoverage of the Poisson model, and slight undercoverage of the other models in settings with high dispersion and short estimation windows. A difference lies in the fact the severity of Poisson undercoverage also increases with higher incidence magnitude. This is a direct consequence of the quadratic mean-variance relationship of NegBin-Q process used to generate the data. We note that no explicit formula for the actual coverage of the Poisson model is available in this setting as (unlike in the NegBin-L case) equation (5) cannot be simplified in a suitable manner.

The empirical distribution of $\hat{R}$ estimates under NegBin-Q, shown in Supplementary Figure S12, is similar to that observed when NegBin-L is the underlying count distribution. Point estimates are comparable across all models, while the standard errors from the Poisson model are located around smaller values than those from the overdispersed models.

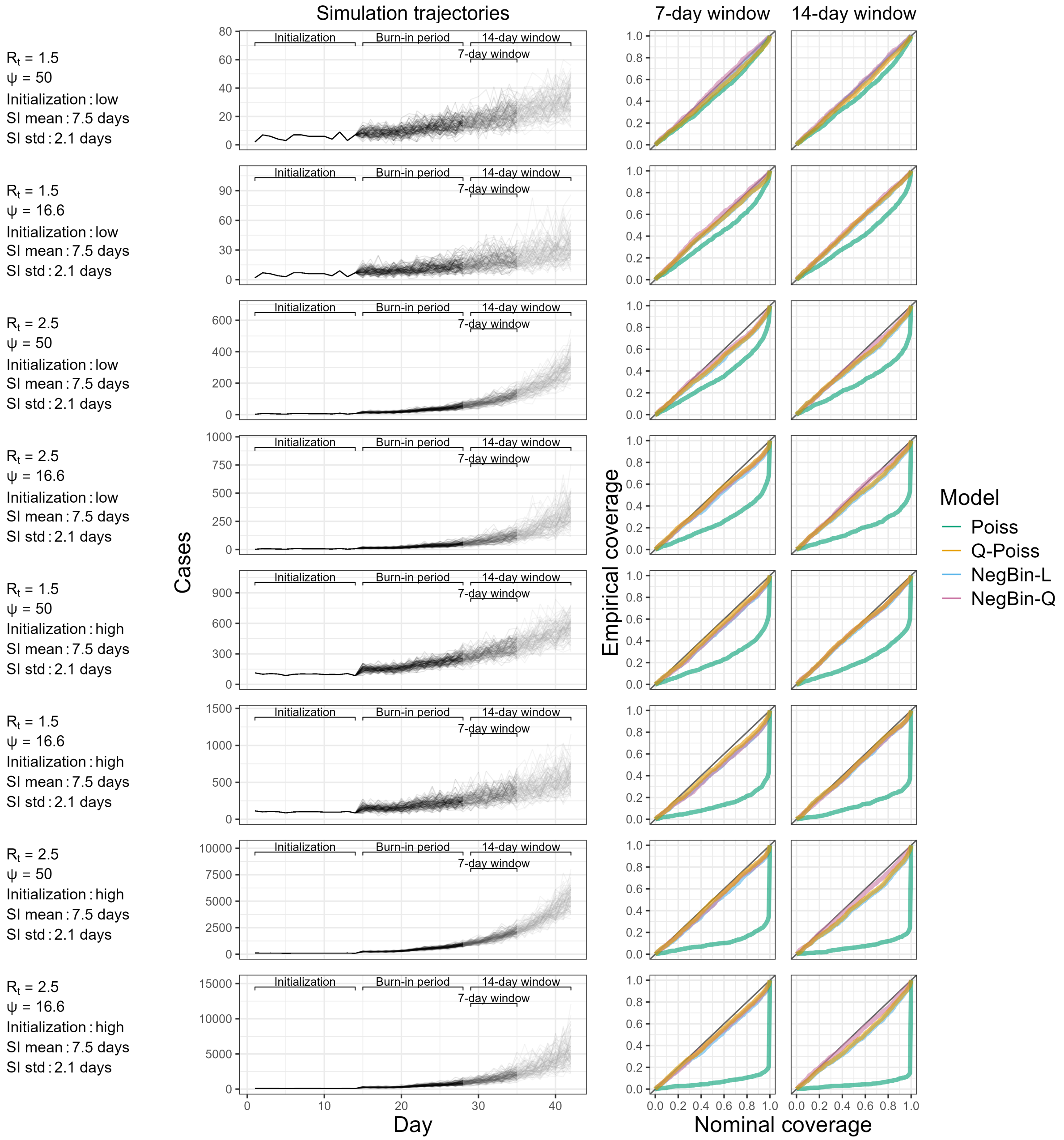

Figure S11: Simulation trajectories generated by the NegBin-Q model with a serial interval typical to RSV and empirical coverage of the four models in scenarios initialized by both low and high values. The left panel shows 1000 incidence trajectories generated from the renewal equation with the NegBin-Q distribution across eight scenarios defined by different parameter combinations specified on the left margin of the figure. The right panels display empirical coverage of the true R for Poisson, quasi-Poisson and negative binomial models across nominal coverage levels, using 7-day (middle column) and 14-day (right column) estimation windows.

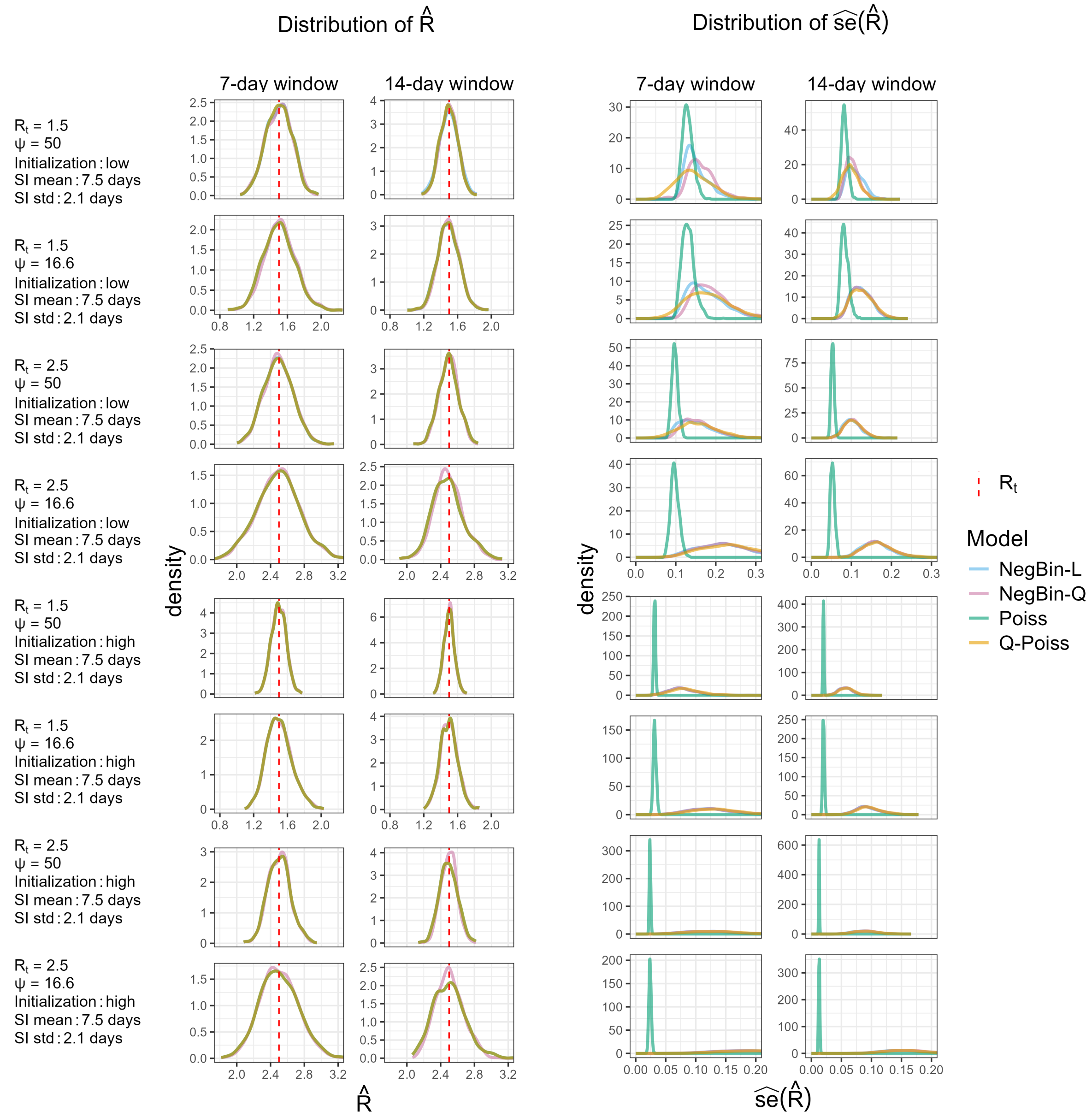

Figure S12: Empirical distribution of the $\hat{R}$ estimates (two left columns) and their standard errors (two right columns) under the NegBin-Q distributional assumptions, low and high initialization values and a serial interval typical for RSV. Different parameter combinations are specified on the left margin of the figure. The red dashed line shows the true value of the effective reproductive number R.

C.2.5 Poisson simulation scenarios

To explore the properties of overdispersed models in settings with no overdispersion present, we conducted four additional simulation scenarios. The parameters were identical to those in Section 2.3 and Supplementary Section C.2.1 ($R \in \{1.5, 2.5\}$ and two different sets of initial values), except that there is no dispersion parameter to vary. Due to the absence of overdispersion, the fitting algorithm of the negative binomial models managed to converge only in roughly one third of the simulation runs. Supplementary Figure S13 displays the generated trajectories and corresponding coverage results. As expected, the empirical and the nominal coverage are aligned best for the true Poisson model. For the quasi-Poisson model, we recognize weak tendencies to undercover. Since the tendency is stronger for the short estimation window, we ascribe this to the effect of small-sample biases. In contrast, the negative binomial models seem to reach higher empirical coverage than the specified nominal levels. This can be explained by the inability of the negative binomial models to handle underdispersion. They detect occasional overdispersed trajectories, while for underdispersed ones, the Poisson estimate is used, which can be seen as a limiting case for the overdispersion getting vanishingly small. As a result, confidence intervals from the negative binomial models are, on average, somewhat wider.

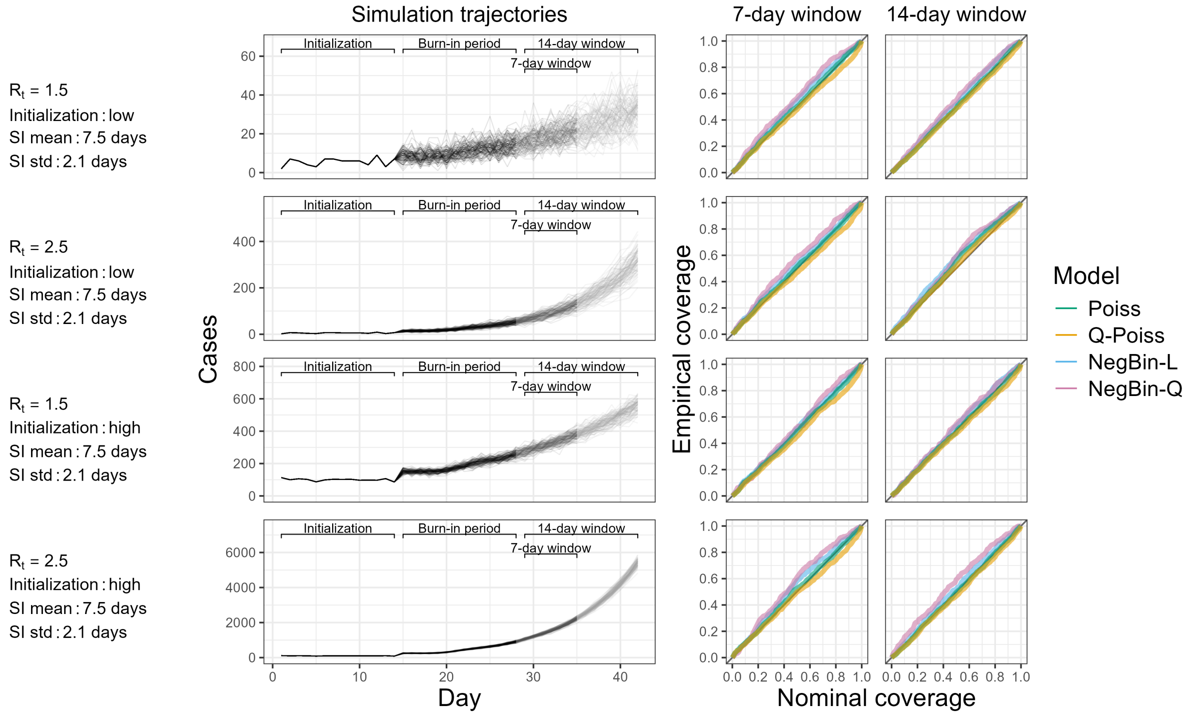

Figure S13: Simulation trajectories generated by the Poisson model with a serial interval typical to RSV and empirical coverage of the four models in scenarios initialized by both low and high values. The left panel shows 1000 incidence trajectories generated from the renewal equation with the Poisson distribution across four scenarios defined by different parameter combinations specified on the left margin of the figure. The right panels display empirical coverage of the true R for Poisson, quasi-Poisson and negative binomial models across nominal coverage levels, using 7-day (middle column) and 14-day (right column) estimation windows.

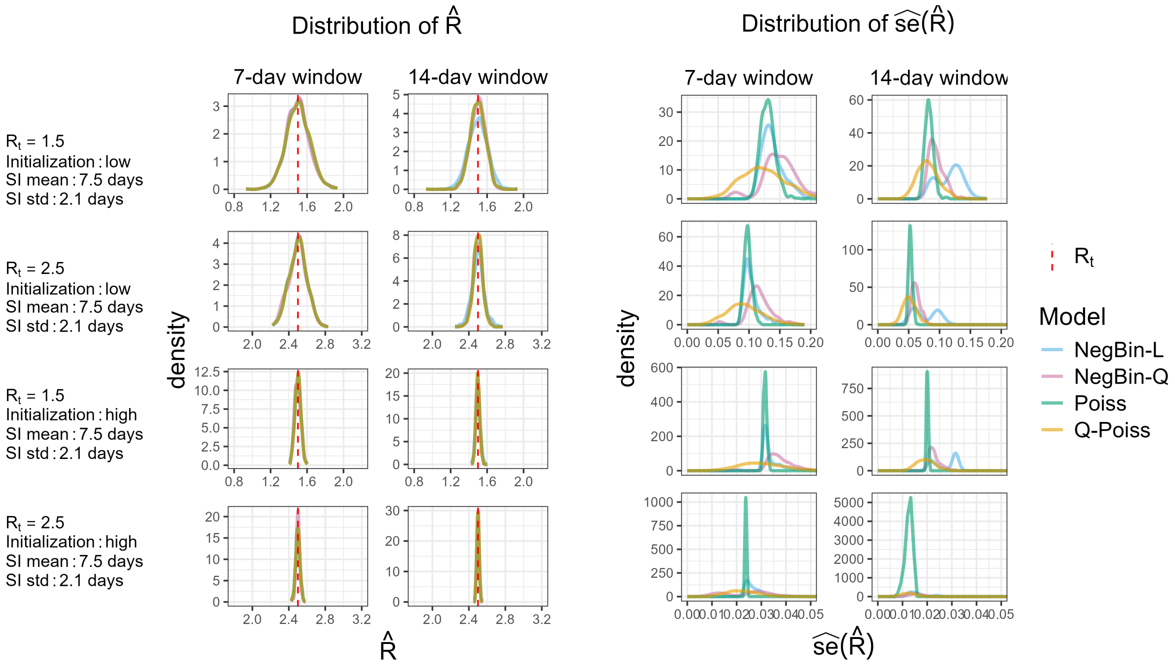

Figure S14: Empirical distribution of the $\hat{R}$ estimates (two left columns) and their standard errors (two right columns) under the Poisson distributional assumptions, low and high initialization values and a serial interval typical for RSV. Different parameter combinations are specified on the left margin of the figure. The red dashed line shows the true value of the effective reproductive number R.

C.2.6 Branching process simulation scenarios

To further illustrate the properties of the Poisson renewal equation model compared to the overdispersed models, we simulate incidence data using a model that is structurally different from a renewal process. In this section, we employ a branching process model in which infections are represented by the number of offspring generated by each individual.

The process is initialized with a fixed number of individuals at times $t = 1, \ldots, 14$. The numbers of initially infectious individuals were set to be (1, 6, 5, 3, 2, 6, 6, 5, 5, 5, 3, 8, 2, 6) Each individual produces a number of offspring that is distributed in calendar time according to the serial interval distribution. Overdispersion enters the process through clustering. Specifically, each individual generates a number of clusters, where the number of clusters follows a Poisson distribution with mean $R/(\gamma - 1)$. Cluster sizes are also Poisson distributed with mean $(\gamma - 1)$, ensuring that the mean and variance of the offspring distribution are R and γR, respectively. All individuals within a cluster share the same serial interval. To obtain the incidence curve, we aggregate all individuals according to the calendar time. The process runs for 28 days, before the estimation window starts and R is constant throughout the whole trajectory.

To add realism, we assume some degree of underreporting, where only a proportion $\kappa < 1$ of all infections is observed. Given the total incidences X_t, the reported incidences X_t^* are independently binomially distributed:

$$X_t^* \overset{\text{ind.}}{\sim} \text{Binom}(X_t, \kappa),$$

and we treat X_t^* as the observable incidence curve.

The generated trajectories of X_t^* are shown in the left panel of Supplementary Figure S15. The serial interval distribution matches that used in Section 2.3. To avoid explosive trajectories that require longer time to generate using the branching process, we selected lower reproductive numbers, $R \in \{1.2, 2\}$. These

values were combined with two levels of offspring overdispersion, $\gamma \in \{1.5, 3\}$. A constant underreporting proportion $\kappa = 0.5$ was assumed across all scenarios.

In Supplementary Figure S15, we can see that the Poisson model again exhibits the worst coverage of all models, which becomes more pronounced in scenarios with higher dispersion. The empirical coverage of the remaining models aligns well with the nominal levels, except for minor fluctuations in panels corresponding to the shorter estimation window.

The empirical distribution of $\hat{R}$ estimates for data generated from the branching process are shown in Supplementary Figure S16. The plots closely resemble those obtained in the simulation scenarios where a negative binomial renewal model served as the data-generating mechanism. Point estimates $\hat{R}$ are similar across all models, while the standard errors from the Poisson model are smaller than those from the overdispersed models.

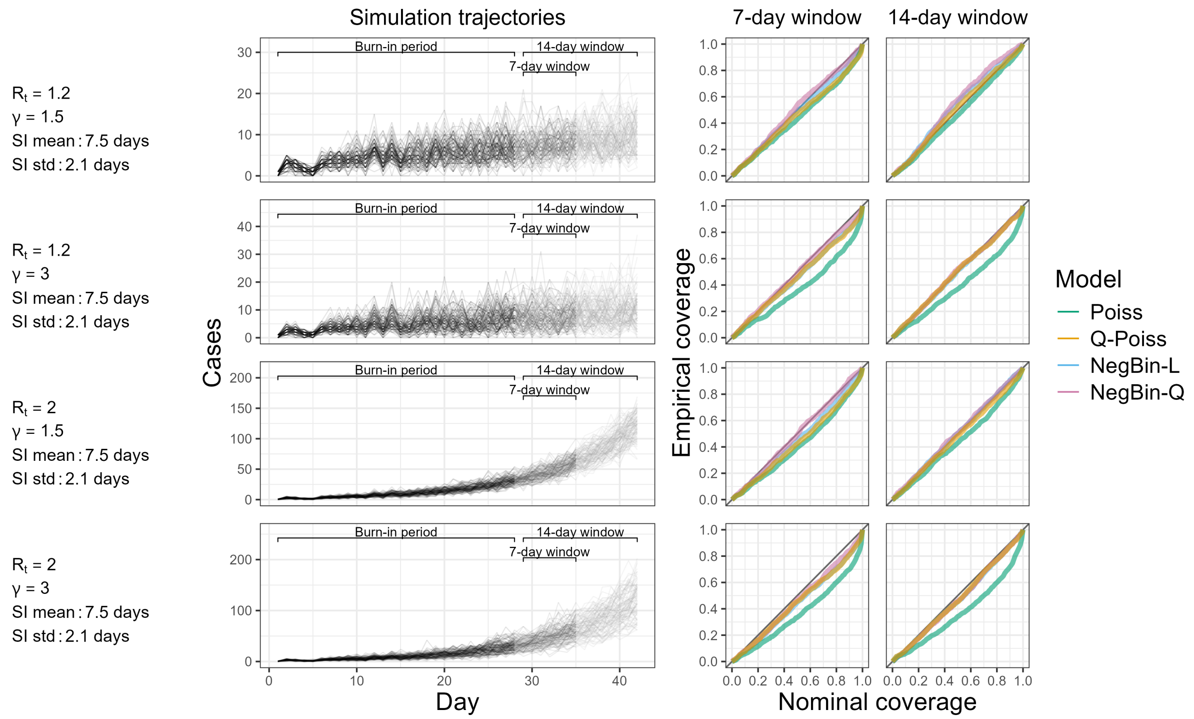

Figure S15: Simulation trajectories generated by the branching process model with a serial interval typical to RSV and empirical coverage of the four models. The left panel shows 1000 incidence trajectories generated from the branching process model across four scenarios defined by different parameter combinations specified on the left margin of the figure. The right panels display empirical coverage of the true R for Poisson, quasi-Poisson and negative binomial models across nominal coverage levels, using 7-day (middle column) and 14-day (right column) estimation windows.

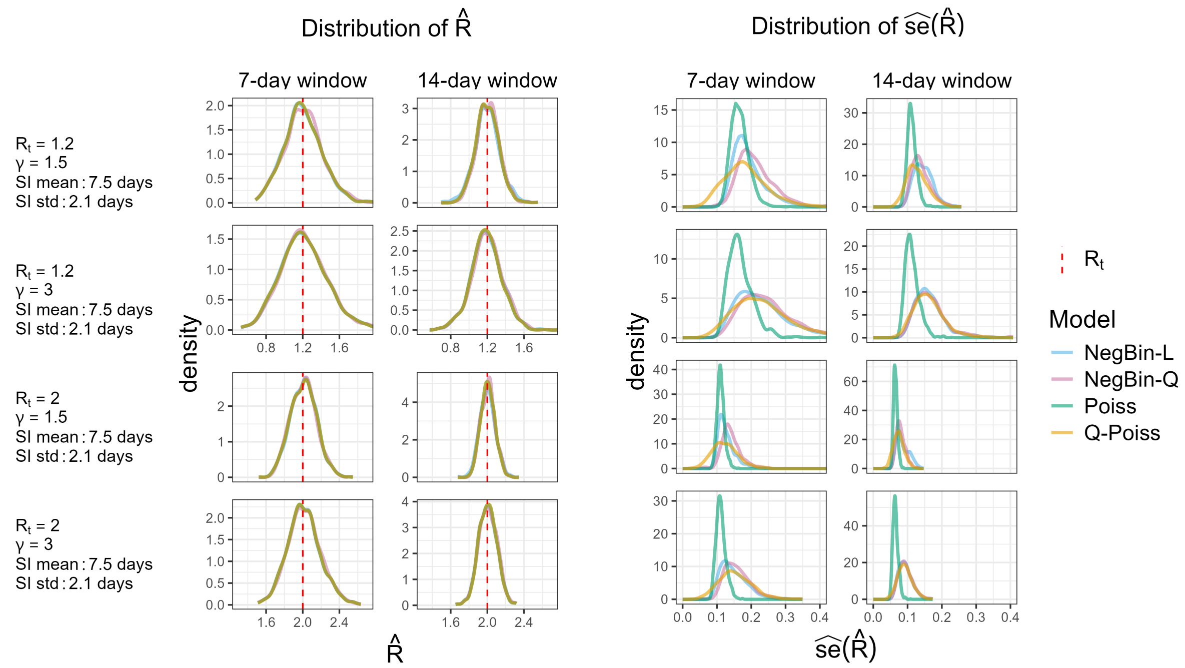

Figure S16: Empirical distribution of the $\hat{R}$ estimates (two left columns) and their standard errors (two right columns) under the Branching process data generating process and a serial interval typical for RSV. Different parameter combinations are specified on the left margin of the figure. The red dashed line shows the true value of the effective reproductive number R.

C.3 Empirical distribution of $\hat{\xi}$ and $\hat{\psi}$

Supplementary Figure S17 shows the empirical distribution of the dispersion parameter estimates across the different simulation scenarios, where NegBin-L is the true data generating process. The estimates in the left column correspond to scenarios discussed in the main text (first four rows) and Supplementary Section C.2.1 (bottom four rows). The right column displays the dispersion parameter estimates from Supplementary Section C.2.2. Note that the plotted values are the reciprocals of ξ. We invert the scales here, because then we can directly compare the values of the NegBin-L dispersion parameter $1/\xi$ and quasi-Poisson dispersion parameter ϕ if we shift the quasi-Poisson estimates to the left by one unit. In other words, ξ and $(\phi - 1)^{-1}$ are on the same scale.

We can notice that for scenarios with low overdispersion, the quasi-Poisson model sometimes suggests values of $\phi < 1$, which corresponds to the simulated trajectories occasionally exhibiting underdispersion. For scenarios with higher dispersion, the estimates are fairly similar. Overall, the estimates are concentrated around the true value with the estimates using the short estimation window (dotted lines) being less accurate.

Figure S18 corresponds to the scenarios described in Section C.2.4 with NegBin-Q being the underlying true distribution. We plot the values of $1/\psi$ here to remain conceptually consistent with Figure S17. Again, the estimates using only the short estimation window seem to be less accurate.

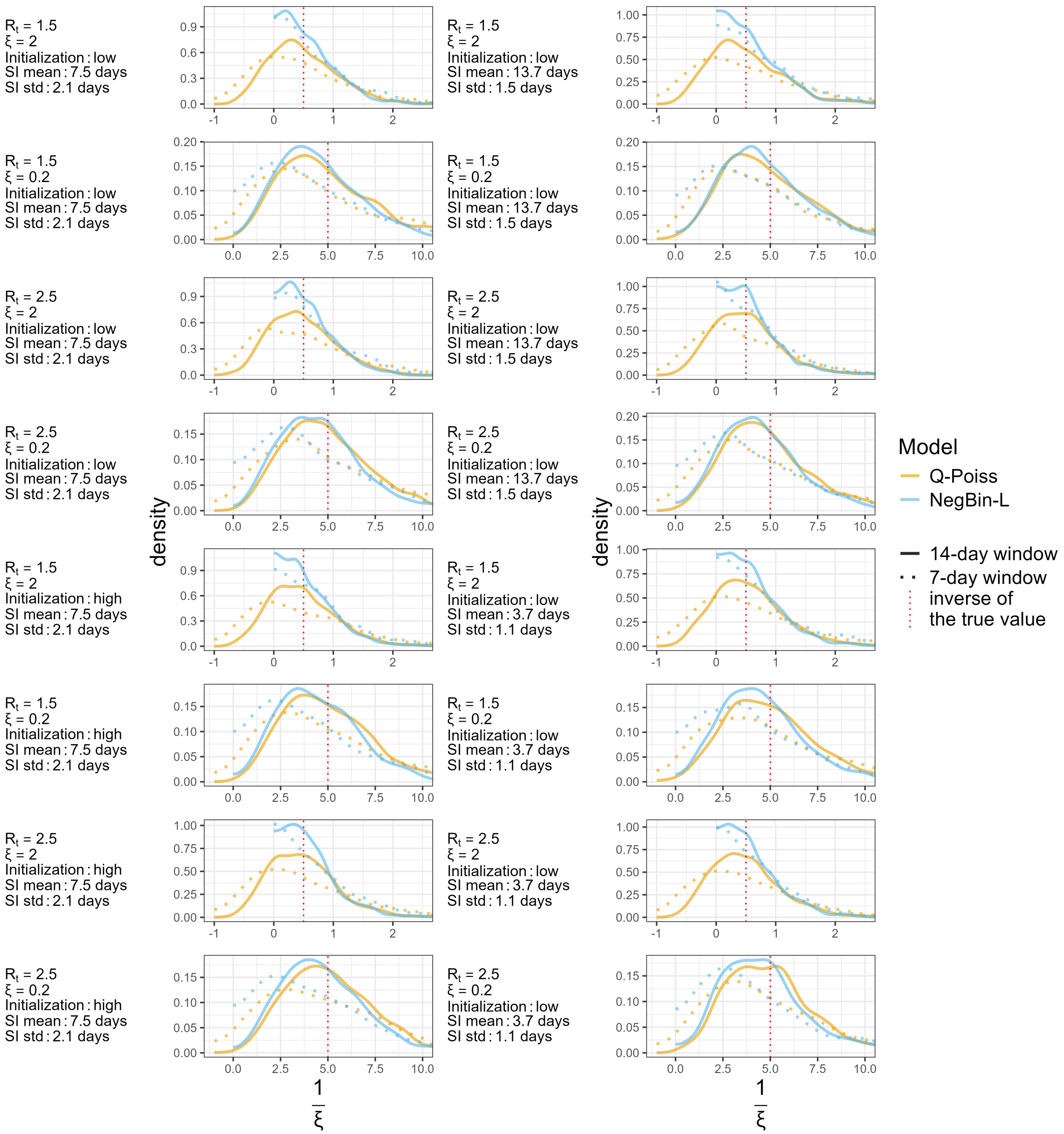

Figure S17: Empirical distribution of the $1/\hat{\xi}$ estimates under the NegBin-L distributional assumptions. Different parameter combinations are specified on the left margin of each panel. The red dashed line shows the true value of the dispersion parameter.

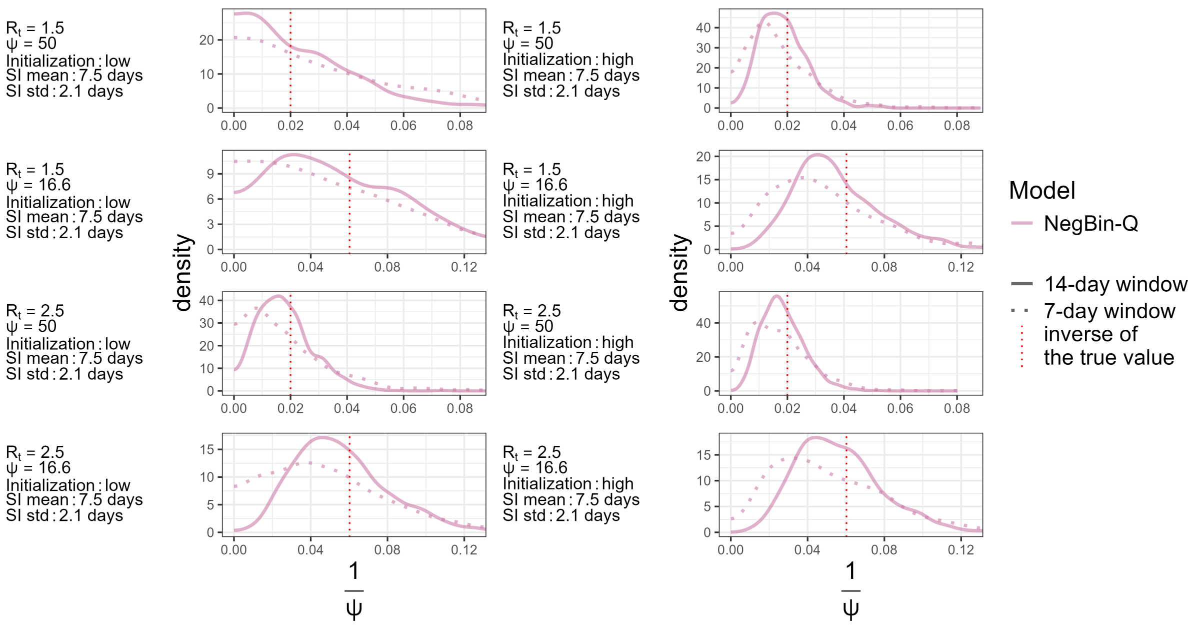

Figure S18: Empirical distribution of the $1/\hat{\psi}$ estimates under the NegBin-Q distributional assumptions. Different parameter combinations are specified on the left margin of each panel. The red dashed line shows the true value of the dispersion parameter.

D Details on case studies

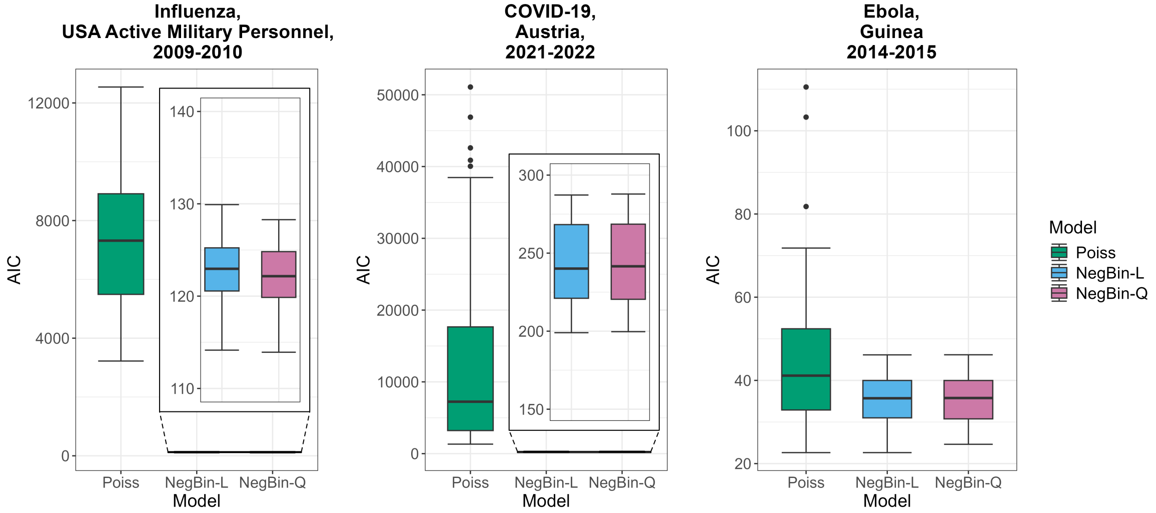

Figure S19: Boxplots of AIC values in the three case studies from Figure 2. In the Influenza and COVID-19 examples, the AIC values corresponding to the negative binomial models are substantially lower than those from the Poison model, which makes the corresponding boxplots visually indiscernible in comparison to the Poisson one. Therefore we zoom in on the negative binomial boxplots in the plot insets.

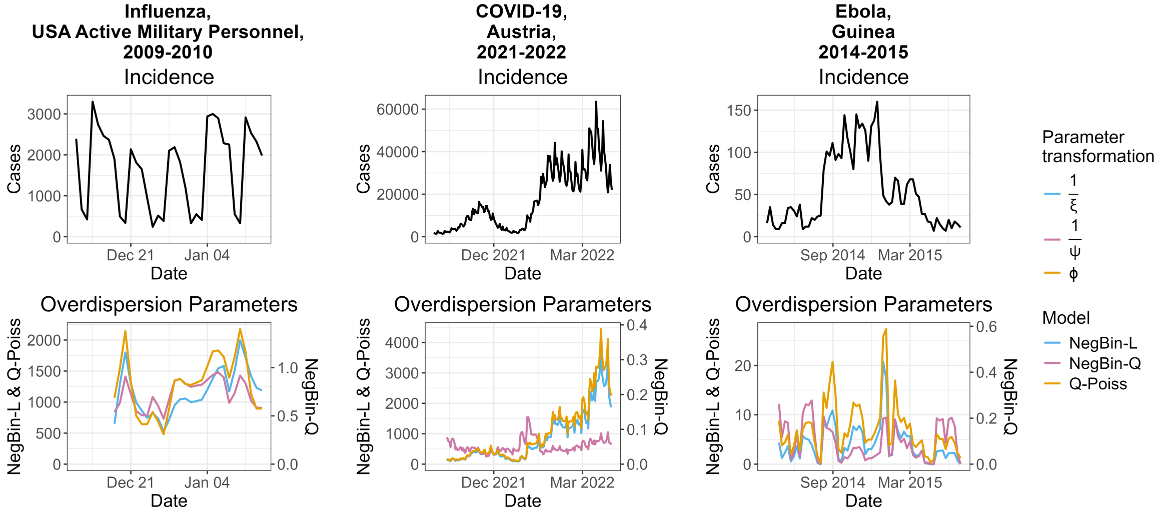

Figure S20: Estimated dispersion parameters in the three case studies from Figure 2. The left vertical axis refers to the quasi-Poisson and NegBin-L models showing values $1/\xi$ and ϕ, while the right vertical axis refers to the NegBin-Q model showing values of $1/\psi$.

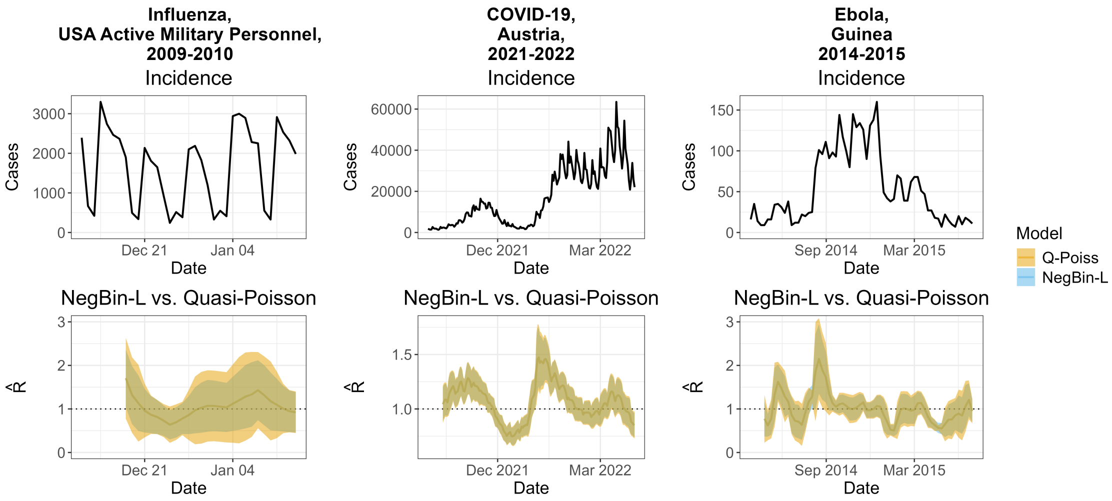

Figure S21: Comparison of R estimates from the quasi-Poisson and NegBin-L models in the case studies from Figure 2.

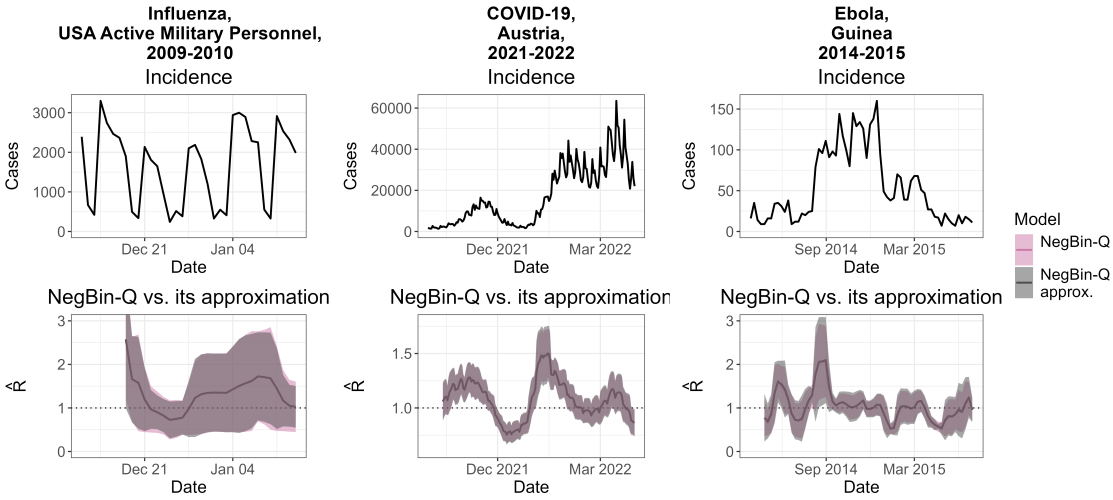

Figure S22: Comparison of R estimates and associated Wald confidence intervals from the NegBin-Q model obtained using the `gamlss` package and approximate formulas (8), (9) and (S12). Agreement is overall close, with minor discrepancies for the Ebola case study.

E Example of renewal equation model estimation in R

We briefly illustrate how standard regression functionality in R can be leveraged to apply each of the presented overdispersed renewal equation models. The following code uses the package `EpiEstim` to construct serial interval distributions and `gamlss` for model fitting.

```
library(gamlss)
# Incidence example taken from the Influenza dataset
incid <- c(2393, 668, 421, 3299, 2742, 2465, 2365, 1899, 494, 337, 2136, 1809,
           1649, 940, 242, 516, 384, 2100, 2187, 1827, 1194, 327, 549, 410)
# Define the serial interval distribution
si <- EpiEstim::discr_si(seq_along(incid) - 1, mu = 3.6, sigma = 1.6)
window_width <- 7 # Estimation window width
# Calculate the covariate Lambda using the incidence and the serial interval
Lambda <- sapply(seq(2, length(incid)),
                 function(ind) sum(si[seq_len(ind)] * incid[seq(ind, 1)]))
# Data frames with the incidence and the covariate Lambda for each window
model_data <- sapply(
  seq_len(length(incid) - window_width),
  function(window) {
    data.frame(
      X = incid[seq_len(window_width) + window],
      Lambda = Lambda[seq_len(window_width) + window - 1], # Lambda shorter by 1
      window = window
    )
  },
  simplify = FALSE
)
# Poisson renewal equation model
mod_pois <- lapply(
  model_data,
  function(df) glm(X ~ Lambda - 1, df, family = poisson(link = "identity"))
)
# Quasi-Poisson renewal equation model
mod_qpois <- lapply(
  model_data,
  function(df) glm(X ~ Lambda - 1, df, family = quasipoisson(link = "identity"))
)
# NegBin-L renewal equation model
mod_negbinl <- lapply(
  model_data,
  function(df) {
    gamlss(X ~ Lambda - 1, family = NBII(mu.link = "identity"), data = df)
  }
)
# NegBin-Q renewal equation model
mod_negbinq <- lapply(
  model_data,
  function(df) {
    gamlss(X ~ Lambda - 1, family = NBI(mu.link = "identity"), data = df)
  }
)
```

References

[1] McCullagh P, Nelder JA. (1989) *Generalized Linear Models, Second Edition.* CRC Press.

[2] Fokianos K, Rahbek A, Tjøstheim D. Poisson autoregression. *Journal of the American Statistical Association*, 104(488): 1430–1439, 2009.

[3] Paul M, Held L, Toschke AM. (2008) Multivariate modelling of infectious disease surveillance data. *Statistics in Medicine*, 27(29):6250–6267.

[4] Doukhan P, Leucht A, Neumann MH. (2022) Mixing properties of non-stationary INGARCH$(1,1)$ processes. *Bernoulli*, 28(1):663 – 688.

[5] Cori et al. (2013) A new framework and software to estimate time-varying reproduction numbers during epidemics. *American Journal of Epidemiology*, 178(9):1505–1512.

[6] Fine PEM. (2003) The Interval between Successive Cases of an Infectious Disease. *American Journal of Epidemiology*, 158(11):1039–1047.

[7] Morgan OW et al. (2010) Household transmission of pandemic (H1N1) 2009, San Antonio, Texas, USA, April–May 2009. *Emerging infectious diseases*, 16(4):631–637.
